# Integrating GWAS, linkage mapping and gene expression analyses reveals the genetic control of growth period traits in rapeseed (*Brassica napus* L.)

**DOI:** 10.1186/s13068-020-01774-0

**Published:** 2020-08-03

**Authors:** Tengyue Wang, Lijuan Wei, Jia Wang, Ling Xie, Yang Yang Li, Shuyao Ran, Lanyang Ren, Kun Lu, Jiana Li, Michael P. Timko, Liezhao Liu

**Affiliations:** 1 Chongqing Engineering Research Center for Rapeseed, College of Agronomy and Biotechnology, Southwest University, Chongqing, 400715 China; 2 Academy of Agricultural Sciences, Southwest University, Beibei, Chongqing, 400715 China; 3 Department of Biology, University of Virginia, Charlottesville, VA 22904 USA

**Keywords:** *Brassica napus*, Growth period traits, GWAS, Linkage mapping, RNA sequencing

## Abstract

**Background:**

*Brassica napus* is one of the most important oilseed crops, and also an important biofuel plant due to its low air pollution and renewability. Growth period are important traits that affect yield and are crucial for its adaptation to different environments in *B. napus*.

**Results:**

To elucidate the genetic basis of growth period traits, genome-wide association analysis (GWAS) and linkage mapping were employed to detect the quantitative trait loci (QTL) for days to initial flowering (DIF), days to final flowering (DFF), flowering period (FP), maturity time (MT), and whole growth period (GP). A total of 146 SNPs were identified by association mapping, and 83 QTLs were identified by linkage mapping using the RIL population. Among these QTLs, 19 were pleiotropic SNPs related to multiple traits, and six (q18DFF.A03-2, q18MT.A03-2, q17DFF.A05-1, q18FP.C04, q17DIF.C05 and q17GP.C09) were consistently detected using both mapping methods. Additionally, we performed RNA sequencing to analyze the differential expression of gene (DEG) transcripts between early- and late-flowering lines selected from the RIL population, and the DEGs were integrated with association mapping and linkage analysis to confirm their roles in the growth period. Consequently, 12 candidate genes associated with growth period traits were identified in *B. napus*. Among these genes, seven have polymorphic sites in the coding sequence and the upstream 2-kb sequence based on the resequencing data. The haplotype BnaSOC1.A05-Haplb and BnaLNK2.C06-Hapla showed more favorable phenotypic traits.

**Conclusions:**

The candidate genes identified in this study will contribute to our genetic understanding of growth period traits and can be used as targets for target mutations or marker-assisted breeding for rapeseed adapted to different environments.

## Introduction

*Brassica napus* (*B. napus*, genome AACC, 2*n* = 38) is one of the most important oilseed crops and an important source of protein-rich livestock feed in the world. At the same time, due to the energy crisis, low air pollution and renewability of rapeseed oil, an increasing number of people regard rapeseed oil as an ideal green energy source. This species is an allopolyploid (AACC) that evolved from an interspecies cross between *Brassica rapa* (*B. rapa*, genome AA, 2*n* = 20) and *Brassica oleracea* (*B. oleracea*, genome CC, 2*n* = 18) approximately 7500 years ago [1]. The species originated in Europe and spread worldwide (to East Asia, Australia, and North America). The growth period of rapeseed can be divided into vegetative growth period (the duration from seedling to flowering), and reproductive growth period (the duration from flowering to maturity). Flowering is a major trait in the plant’s growth period, as it represents the transition from the vegetative stage to the reproductive stage. Flowering time plays an indispensable role in adaptation to specific environments. It has been reported that the growth stages show high correlations with plant height, pod number and seed yield [2]. Therefore, understanding the genetic network underlying growth period traits provides a theoretical and practical basis for developing new cultivars adapted to different geographical environments.

Linkage mapping based on bi-parental populations has been widely applied to detect QTLs for flowering time traits in *B. napus* [3–9]. Long et al. [4] identified 36 significant level (SL-QTL) and 6 micro real QTL (MR-QTL) in the doubled haploid (DH) population (Tapidor × Ningyou7) and its derived F2 population. Raman et al. [6] identified at least 20 flowering time loci localized on ten different chromosomes in the SASDH DH population (Skipton × Ag-Spectrum), which explained 2.4–28.6% of phenotypic variation. Raman et al. [7] further constructed an integrated genetic linkage map of the SASDH population and identified QTLs for flowering time accounted for up to 40.2% of genetic variation. Liu et al. [8] found 22 QTLs for days to flowering (DIF) in the spring-type DH population under multiple spring environments. Four major QTL were located on A7, C2, and C8, and explained 10.0–46.5% of phenotypic variation. Li et al. [10] used DH populations to localize QTL for flowering time in oilseed rape under winter, semi-winter, and spring ecological conditions, and identified 55 consistent QTL, including 12 environmentally stable QTL and 43 environmentally specific QTL.

Genome-wide association studies (GWAS) using natural populations provide higher mapping resolution than linkage mapping based on bi-parental segregating populations and have greater cost-effectiveness. Based on the GWAS website (https://bigd.big.ac.cn/gwas/), approximately 8373 associations related to 19 growth period traits have been identified across six crops (cotton, maize, rapeseed, rice, sorghum, and soybean) using GWAS technology [11]. Among these associations, 198 associations for seven growth period traits were identified in rapeseed. Xu et al. [12] found 41 SNPs correlated with flowering time with a diversity panel comprising 523 *B. napus* cultivars, and 12 SNPs were consistent with previously identified QTLs. Niklas et al. [13] found six QTLs for beginning of flowering (BOF) and one QTL for end of flowering (EOF) in 405 *B. napus* inbred lines. Zhou et al. [14] identified 131 SNPs strongly linked to four earliness traits (initial flowering day, maturity time, final flowering day and flowering period), 40 of which fell into or were physically close to published flowering time SNPs. Wei et al. [15] detected 12 SNPs significantly associated with flowering time using 3,318,6 high-quality SNPs from 327 accessions. From the above results, it can be seen that these studies focused mainly on the initial flowering time. Other growth period traits, such as final flowering stage (DFF), flowering period (FP) and maturity time (MT), have rarely been reported by mapping methods.

RNA sequencing has been widely applied to examine differences in global gene expression with the benefit of high sensitivity and is more cost-effective than microarray analysis. However, transcriptome analysis often obtained a large number of differentially expressed genes (DEGs) between samples. Therefore, the integration of DEGs with GWAS or linkage mapping has been considered to be an effective way to identify candidate genes related to complex traits [16–19].

In this study, using a panel of 588 accessions, we carried out a GWAS for five growth period traits, the number of days to initial flowering (DIF), the number of days to final flowering (DFF), flowering period (FP), maturity time (MT), and growth period (GP). Furthermore, the SNPs associated with DIF, DFF, FP, MT and GP were detected in the recombinant inbred line (RIL) population. In addition, we performed transcriptomic analysis of the leaves of early-flowering and late-flowering accessions at different developmental stages (vegetative and reproductive). The main objectives of our study are: (1) to dissect the genetic mechanism of growth period traits by GWAS using a natural population and QTL mapping using an RIL population derived from GH06 and P174; (2) to dissect the molecular mechanism of floral development by characterizing the DEGs of leaves for early-flowering and late-flowering lines using RNA sequencing technology; and (3) integrating the QTLs and DEGs to identify candidate genes that control growth period traits for further verification.

## Results

### Phenotypic variations, correlations, and ANOVA for growth period traits

**Fig. 1:**
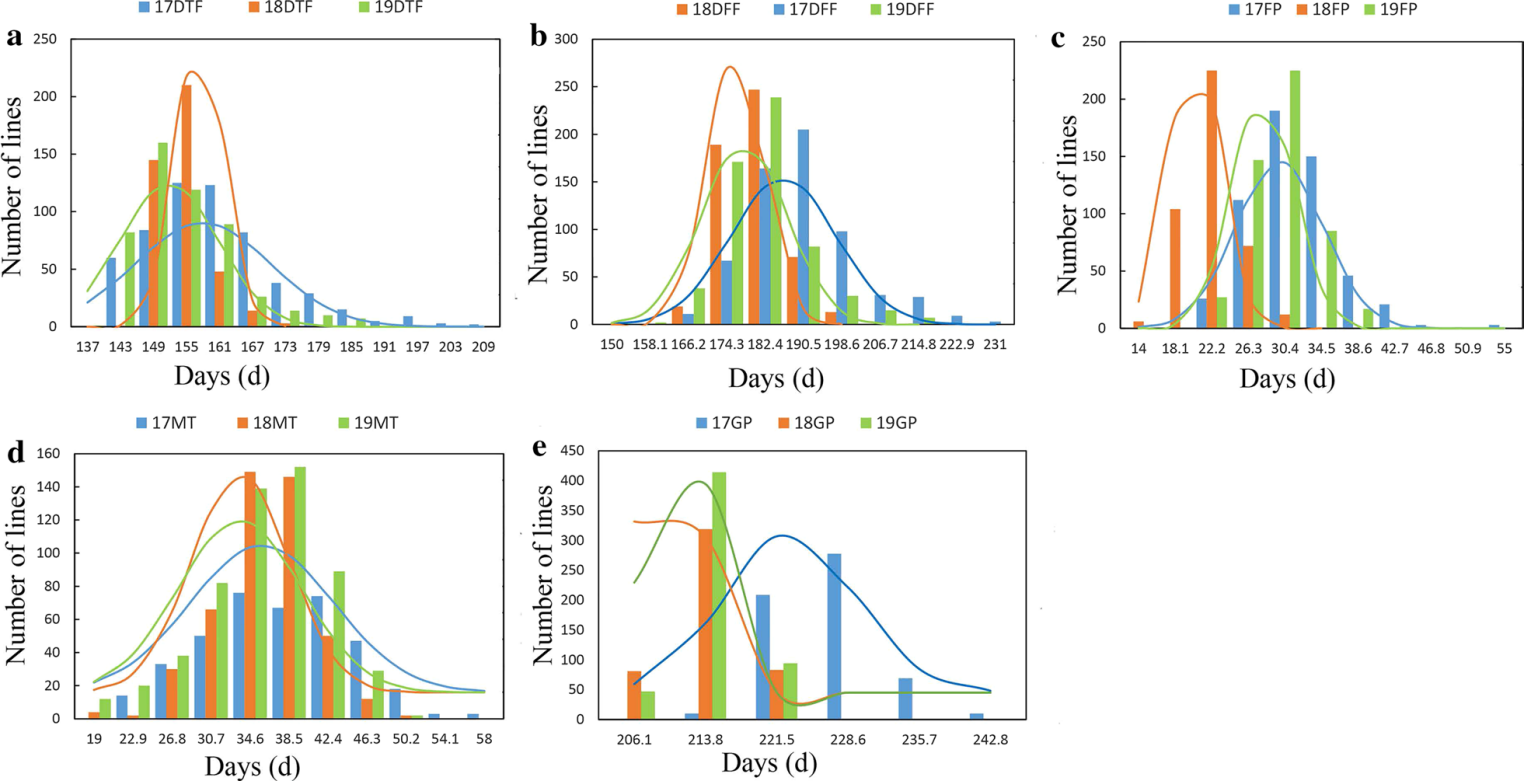
Frequency distribution of five growth period traits in GWAS population in 3 years (2017, 2018 and 2019)

Five growth period traits (DIF, DFF, FP, MT and GP) of 588 rapeseed lines were investigated in 3 years. Significant variation among the genotypes for five growth period traits was observed: for instance, DIF ranged from 138 to 207 days with an average of 157.98 days in 2017, from 149 to 174 days with an average of 157.34 days in 2018, and from 137 to 189 days with an average of 151.87 days in 2019 (Table 1). The coefficient variation (CV) of FP and MT was the highest (over 10%) followed by DIF and DFF, and GP was the lowest. In addition, the phenotypic frequency distributions of five traits in the 3 years all showed approximately continuous and normal distributions, which showed that the group was suitable for association analysis (Fig. 1).To test the effects of genotype (G), environment (E) and their interactions (G × E), we conducted analysis of variance (ANOVA) for each trait (Additional file 1: Table S1a). Significant variations were observed among environments and genotypes for all five traits. The DFF has the highest broad-sense heritability (*h*^2^) of 92.66%, the FP has relatively low heritability (74.11%). Overall, these findings indicated that all five traits were stably inherited.

**Table 1:** Phenotypic variation of five growth period traits in three environments

Environment	Trait	Range (days)	Mean (days)	SD	Variance	CV (%)	Kurtosis	Skewness
17CQ	DIF	138–207	157.98	12.29	150.95	7.78	1.23	0.98
	DFF	157–229	186.28	11.12	123.59	5.97	1.12	0.70
	FP	20–54	29.81	4.89	23.92	16.41	2.30	0.87
	MT	20–56	36.82	6.80	46.23	18.47	− 0.19	− 0.04
	GP	182–272	222.57	7.08	50.07	3.18	10.09	− 0.03
18CQ	DIF	149–174	157.34	4.43	19.66	2.82	1.19	0.98
	DFF	163–197	176.15	6.07	36.80	3.44	0.50	0.66
	FP	14–30	20.27	2.93	8.60	14.47	0.51	0.40
	MT	18–50	33.65	4.86	23.65	14.45	0.34	− 0.23
	GP	204–219	209.82	3.23	10.44	1.54	− 0.84	0.33
19CQ	DIF	137–189	151.87	8.94	79.97	5.89	1.18	1.87
	DFF	147–212	177.99	9.04	81.78	5.08	0.94	1.82
	FP	19–40	27.93	3.51	12.33	12.57	0.31	0.26
	MT	11–50	33.54	6.19	38.32	18.46	− 0.60	0.45
	GP	200–220	210.87	3.17	10.06	1.50	− 0.24	0.53

*17CQ*17 Chongqing, *18CQ* 18 Chongqing, *19CQ* 19Chongqing, *SD* standard deviation, *CV* coefficient of variation, *DIF* days to initial flowering, *DFF* days to final flowering, *FP* flowering period, *MT* maturity time, *GP* growth period

To determine whether there are any relationships between five growth period traits in *B. napus*, the average value of 3 years for each trait was used for correlation analysis (Table 2). DIF showed a significant positive correlation with DFF and GP, but was negatively correlated with FP and MT. DFF was significantly correlated with FP, MT, and GP with correlation coefficients of 0.148, − 0.664 and 0.760, respectively. It can be concluded that a shorter flowering time corresponds to a shorter growth period and a longer maturity time.

**Table 2:** Correlation analysis of five growth period-related traits of GWAS population

Traits	DIF	DFF	FP	MT
DFF	0.705^a^			
FP	− 0.302^a^	0.148^a^		
MT	− 0.626^a^	− 0.664^a^	− 0.004	
GP	0.581^a^	0.760^a^	− 0.016	− 0.316^a^

^a^Correlation is significant at the 0.01 level (2-tailed). DIF, days to initial flowering; DFF, days to final flowering; FP, flowering period; MT, maturity time; GP, growth period

### Genome-wide association analysis for growth period traits in 3 years

Through a set of processes of library construction, paired-end sequencing and SNP calling, a series of 3,856,91 highly consistent and locus-specific SNPs (minor allele frequency > 0.05 and call frequencies > 0.9) were retained for the following analysis. Additional file 2: Fig. S1 shows the density distribution of SNP markers on different chromosomes. The 3,856,91 SNPs were covered and unevenly distributed on all 19 *B. napus* chromosomes.

To avoid false-negative associations, three general linear models (GLM), naïve, PCA and Q models, and three mixed linear models (MLM), K, Q + K and PCA + K models were chosen to evaluate the effects of population structure (Q, PC) and relative kinship (K). According to the Q–Q plots of the six models, the MLM model can control false positives well for each trait in 3 years (Additional file 3: Fig. S2). To minimize the effect of environmental variation, best linear unbiased predictor (BLUP) value for each line were also calculated for each trait using the R package lme4 [20]. Therefore, the MLM models were conducted for GWAS of five growth period traits with 3 years’ data and BLUP values. We mainly focused on the QTLs detected in at least two environments. In total, 146 SNP loci significantly associated (− log10(P) ≥ 5.58) with five growth-related traits were identified, including 13 related to DIF, 23 to DFF, 6 to FP, 19 to MT and 85 to GP for the whole panel of accessions (Additional file 3: Fig. 2 and Additional file 4: Table S2).

**Fig. 2:**
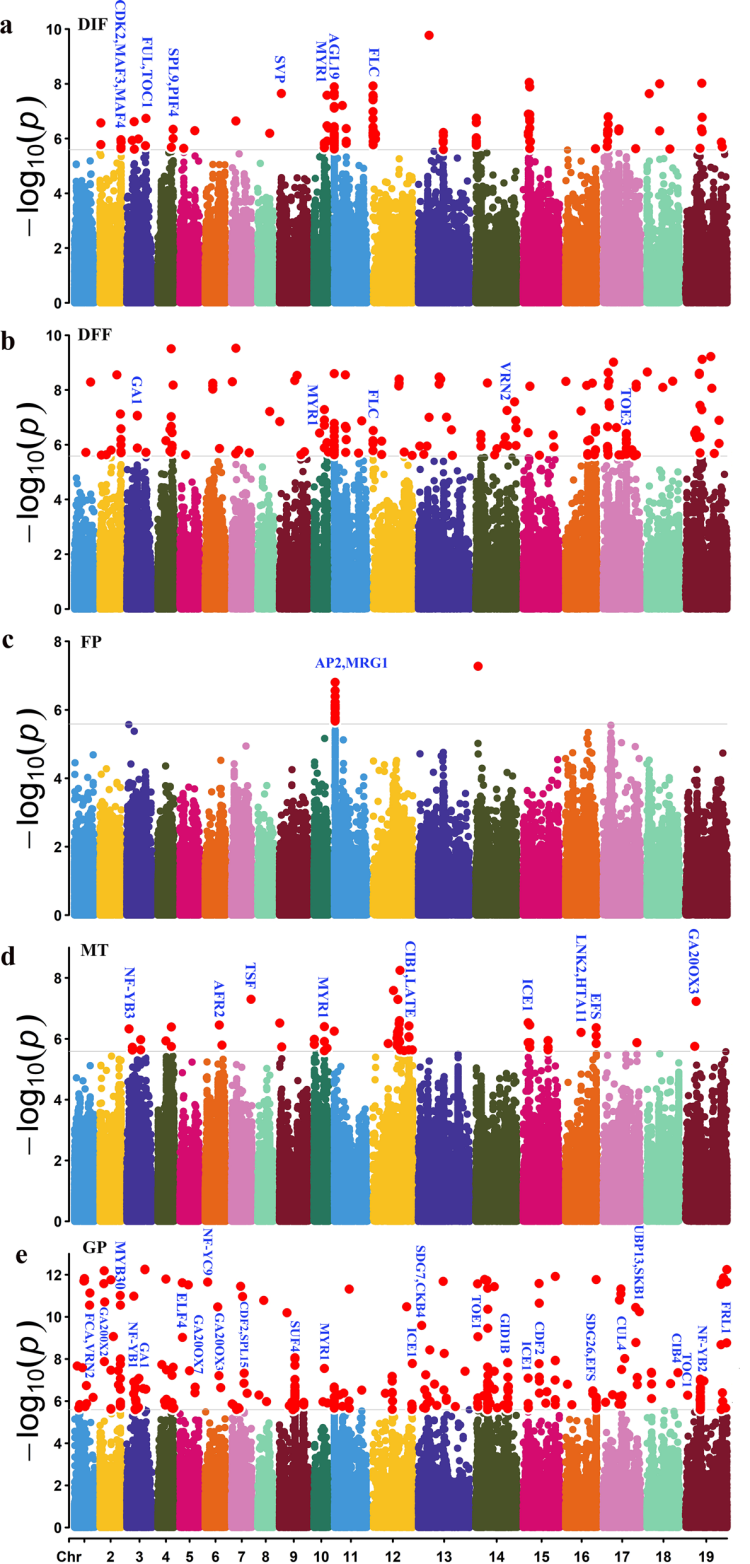
The Manhattan plots for five growth period traits using BLUP value. Different colors represent different chromosomes of *B. napus* (A1–A10, C1–C9). The solid horizontal line (in grey color) signifies the threshold for significant associations [− log10(1/385,691) = 5.58]. Significant SNPs above the threshold line on all chromosomes are highlighted in red. The position of candidate flowering genes that located in the vicinity of the significant SNP is shown

### Identification of candidate genes for growth period traits in *B. napus*

Among the 146 SNP loci significantly associated with growth period traits, 60 SNPs were divided into 19 genomic regions using a haplotype block estimation, with sizes ranging from 7 bp to 270.31 kb, while the remaining 86 SNP loci were not present in the LD blocks (Additional file 5: Table S3). Then, we obtained the genes within the same LD block or within 300 kb to either side of the significant SNPs using *B. napus* ‘Darmor v4.1’ as the reference genome. Finally, a total of 101 candidate genes were identified as orthologous to *Arabidopsis* flowering genes reported in the Flowering Interactive Database (FLOR-ID), most of which were involved in six flowering pathways of aging, autonomous pathway, vernalization, photoperiod, GA, and circadian clock; other genes functioned as flower development and meristem identity and flowering time integrator (Additional file 6: Table S4). The flowering time integrators *FLOWERING LOCUS C* (*FLC*) control the transition from vegetative to reproductive meristem by integrating the signals from six pathways and then precisely regulating the expression of specific flower meristem identity genes *APETALA2* (*AP2*) and *FRUITFULL* (*FUL*) [21].

**Fig. 3:**
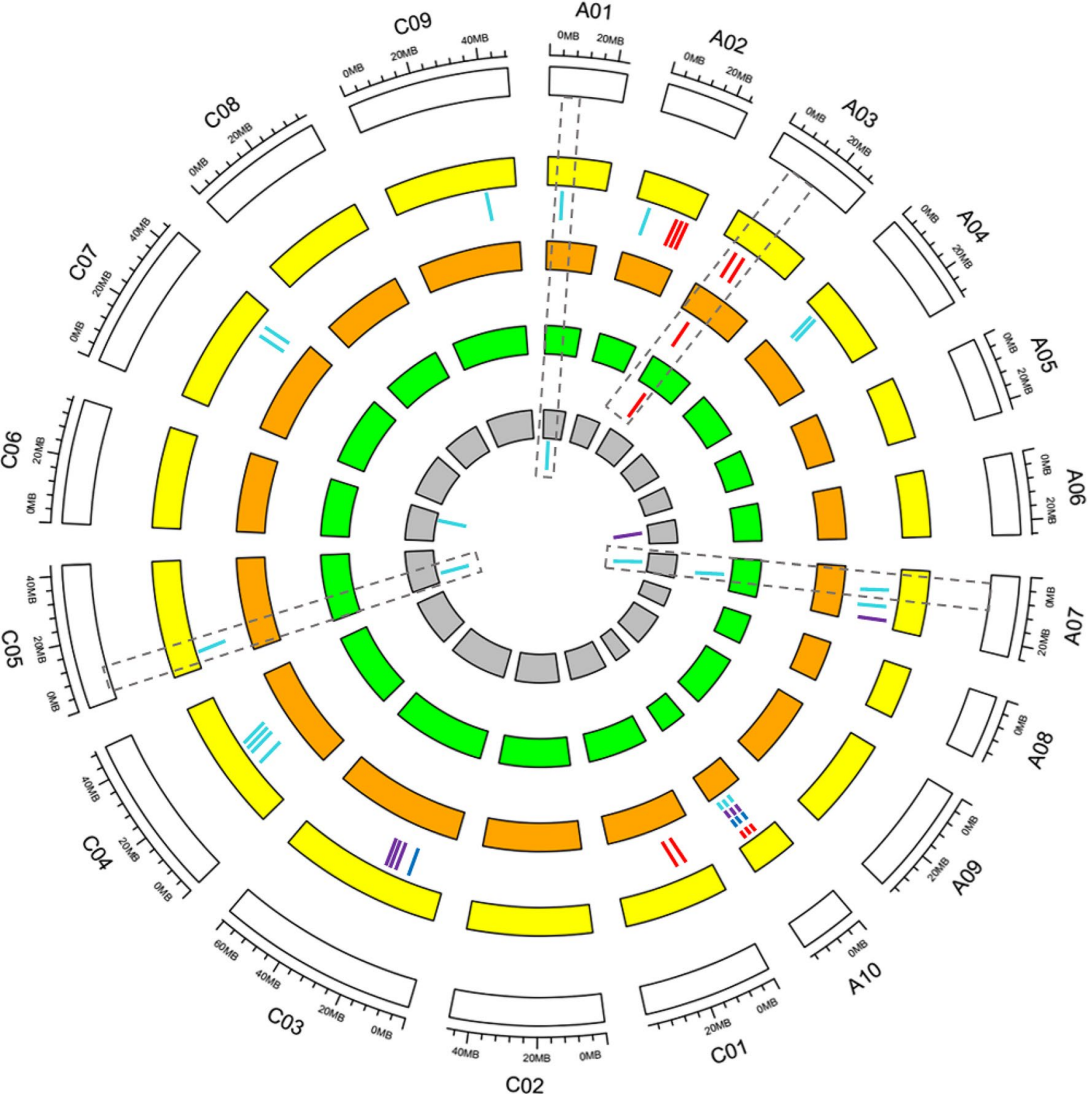
Distribution of consensus QTLs for five growth period traits among different populations. From inside to outside, the four inner circles with different color represent four populations (Raman, Udall, Zhou and Wang, respectively), and short bars with color within the four inner circles represent SNPs identified in different populations (red bars, QTLs for DIF; blue bars, DFF; purple bars, MT; bright green bars, GP). The dotted lines indicate that the SNP loci detected in different populations are co-localized. The blocks at the outermost circle represent the 19 genetic linkage groups

By comparing the SNP regions, 28 SNP loci (within 300 kb) associated with growth period traits, were located in or near the QTL regions identified in previous studies: 23 were reported by Raman et al., seven were reported by Wang et al. and four were reported by Zhou et al. [3, 14, 22, 23] (Additional file 5: Table S3 and Fig. 3). Among these loci, S1_5075025, S2_5719334 and S15_5922896 were reported in two studies, and S3_13708544 and S7_10803897 were detected simultaneously in three studies. At the same time, the candidate genes we identified in this study were also reported in previous studies [14, 22–28]. The flowering time integrator (*BnaFLC.C02*, *BnaFUL.A03* and *BnaSVP.A09*) were reported in three or more previous studies (Additional file 5: Table S3). Overall, the comparison of QTLs and candidate genes further validates the reliability of the GWAS mapping and help us mine the novel QTL.

### QTL linkage mapping of growth period traits in 2 years

The RIL population (GH06 × P174) grown in 2017 and 2018 was evaluated for DIF, DFF, FP, MT and GP phenotypes and then used for QTL analysis. The flowering time of GH06 was later than that of P174 in the two investigated environments. A wide range of variation (Table 3) and the normal phenotypic distribution for the growth period traits (Additional file 7: Fig. S3) were observed in the RIL population, indicating the quantitative inheritance suitable for QTL mapping. ANOVA was performed on the phenotypic data from 2 years, and *h*^2^ ranged from 49% (DFF) to 53% (GP) (Additional file 1: Table S1b).

**Table 3:** Phenotypic variation of five growth period traits for the two parents and derived RIL populations in two environments

Environment	Trait	Parents		RIL population							
		GH06	P174	Range (days)	Mean (days)	SD	Median	Variance	CV(%)	Kurtosis	Skewness
17CQ	DIF	147	135	131–161	143.72	6.33	144	40.12	4.41	0.08	− 0.71
	DFF	171	171	157–186	173.85	5.04	174	25.44	2.90	− 0.60	0.45
	FP	24	36	21–39	30.40	3.82	30	14.61	12.57	0.16	− 0.52
	MT	43	41	30–54	40.41	4.10	40	16.78	10.14	0.52	0.73
	GP	214	212	205–220	214.24	3.03	214	9.21	1.42	− 0.36	0.00
18CQ	DIF	155	149	148–160	152.90	2.59	153	6.70	1.69	0.28	0.02
	DFF	170	168	165–179	170.84	2.94	171	8.63	1.72	0.25	− 0.42
	FP	15	19	13–23	18.19	2.18	18	4.76	11.99	0.11	− 0.23
	MT	39	35	25–43	34.27	3.26	34	10.61	9.51	0.04	− 0.07
	GP	209	203	201–212	205.14	3.07	204	9.44	1.50	0.63	− 0.72

17CQ, 17 Chongqing; 18CQ, 18 Chongqing; SD, standard deviation; CV, coefficient of variation; DIF, days to initial flowering; DFF, days to final flowering; FP, flowering period; MT, maturity time; GP, growth period

**Fig. 4:**
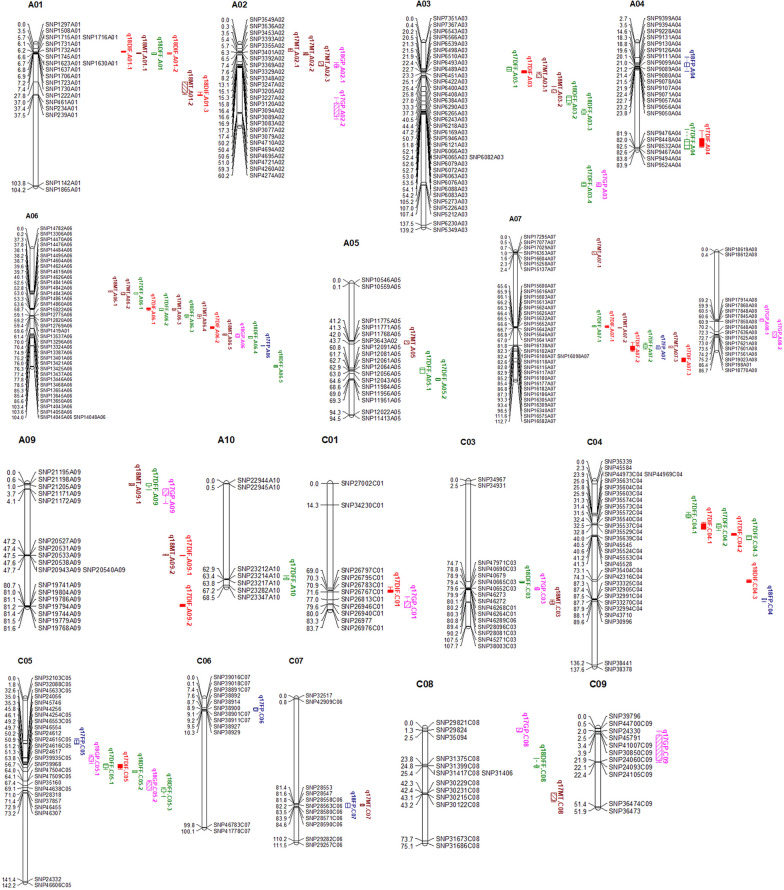
The localization of significant QTLs for five growth period traits on the high-density SNP genetic map in the RIL population. Different colored markers represent different traits (red bars: DIF; green bars: DFF; blue bars: FP; khaki bars: FP and purple bars: GP). Map distances are given in cM and displayed using the MapChart. For simplicity, only show the markers in the QTL confidence intervals, along with the terminal two markers at each end of the QTL-containing chromosomes, the detailed marker information on the genetic linkage map was referred to Liu et al.2013 [65]

The LOD score plots across 19 linkage groups are shown in Additional file 8: Fig. S4. A total of 17, 25, 7, 21 and 13 QTLs for DIF, DFF, FP, MT and GP were detected in 2 years and were located on all *B. napus* chromosomes except C02 (Additional file 9: Table S5 and Fig. 4). These QTLs for DIF, DFF, FP, MT and GP explained ~ 17.48%, ~ 15.92%, ~ 7.95%, ~ 18.30% and ~ 9.43% of the phenotypic variance, and the additive effect varied from − 0.43 to 2.30, − 1.12 to 1.79, − 0.83 to 0.71, − 2.09 to 1.13, and from − 0.70 to 1.10, respectively. The QTLs have the overlapped confidence intervals with the same direction of additive effect are considered to be the same QTL. Among the 83 QTLs, 14 QTLs were associated with at least two traits. The QTL q18DIF.A01-2, q18DFF.A01 and q18MT.A01-1 have the same QTL region and explained the highest total phenotypic variation of each trait. However, the additive values of DIF and DFF were positive, and the additive value of MT was negative, which indicates that the QTL from GH06 increased DIF and DFF, while the QTL from P174 increased MT. The QTL q17DIF.A06-1, q17DFF.A06-2, q17MT.A06-3 and the QTL q17DIF.A07-2, q17DFF.A07-2, q17FP.A07, q17MT.A07-3 also has the same region and opposite additive effect. These results indicate that the same QTL may have opposite effects on the different traits. Based on the rapeseed genome annotation and the physical locations of these QTL regions, a total of 74 *Arabidopsis* flowering homolog genes were identified (Additional file 10: Table S6).

### Identification of DEGs for floral transition and flower development by RNA-sequencing

To identify candidate DEG transcripts controlling flowering time, we sequenced four RNA samples from leaves of early-flowering cultivar 18Z134 and late-flowering cultivar 18Z88 sampled at vegetative and reproductive development stages (EV, ER, LV and LR). Every sample had two replicates. The flowering time differences between 18Z134 and 18Z88 are shown in Additional file 11: Table S7. The total reads, mapped and unique mapped reads to the reference *B. napus* genome are shown in Additional file 12: Table S8. After removing low-quality sequences, 34,862,287 (86.32%)–37,786,256 (90.79%) clean reads were successfully mapped to the genome using TopHat. Of these clean reads, 33,089,439 (81.93%)–38,684,877 (85.93%) were uniquely mapped.

Two types of comparisons were performed: (1) to identify DEGs between the two extreme lines at each development stage: LR vs. ER and LV vs. EV; (2) to identify expression changes between different stages in each line: EV vs. ER and LV vs. LR. The false discovery rate (FDR) ≤ 0.05 and absolute value of |log2 (fold change) | ≥ 1 were used as thresholds to judge the DEGs between the two groups. In total, 3727 DEGs were identified in LR vs. ER (2421 upregulated, 1306 downregulated), 2327 DEGs were identified in LV vs. EV (1557 upregulated, 770 downregulated), 3750 DEGs were identified in EV vs. ER (2135 upregulated, 1615 downregulated), and 2038 DEGs were identified in LV vs. LR (1281 upregulated, 757 downregulated). In addition, 1662 and 683 DEGs were common to LR vs. ER/LV vs. EV and EV vs. ER/LV vs. LR, respectively (Additional file 13: Fig. S5 and Additional file 14: Table S9).

### GO and KEGG analysis of differentially expressed genes

As the first criterion, we analyzed DEGs between vegetative and reproductive stages (EV vs. ER and LV vs. LR) to identify the key phase transition-associated genes. The 683 common DEGs between EV vs. ER and LV vs. LR were subjected to an enrichment analysis for GO annotation terms. The top 20 significantly enriched GO terms are shown in Additional file 15: Fig. S6a and Additional file 16: Table S10a. Among these terms, the salicylic acid biosynthetic process (GO:0009697), response to cold (GO:0009409), negative regulation of floral organ abscission (GO:0060862), intracellular auxin transport (GO:0080162) and long-day photoperiodism, flowering (GO:0048574) were involved in the phase transition process. To further determine the metabolic pathways in the phase transition process, we performed KEGG enrichment analysis (Additional file 15: Fig. S6b and Additional file 16: Table S10b). Circadian rhythm–plant (ko04712) and plant hormone signal transduction (ko04075) were significantly enriched and participate in plant development and flowering processes.

As the second criterion, we searched for DEGs between early-flowering and late-flowering lines that result in flowering time variation. The 2327 DEGs between LV vs. EV and 3727 DEGs between LR vs. ER were subjected to GO and KEGG enrichment analysis (Additional file 17: Fig. S7). DEGs in the circadian rhythm were enriched in both GO and KEGG analysis between LR vs. ER, suggesting that the different expression of genes involved in circadian rhythm could result in flowering time difference.

### Identification of transcription factors and hormone-related flowering genes 

In this study, 50 transcription factor (TF)-encoding DEGs, including the basic helix–loop–helix (bHLH; five members), ERF (eight members), MIKC_MADS (eight members), NAC (three members) and WRKY (four members), were identified in the early- and late-flowering lines at two developmental stages. Additional file 18: Fig. S8a shows the overall expression trend in the four samples, and most TF families were significantly upregulated in the reproductive stages.

In addition, 20 genes in the ABA signaling pathway, 23 genes in the auxin signaling pathway, five genes in the GA signaling pathway, five genes in the cytokinin signaling pathway, four genes in the JA signaling pathway and five genes in the SA signaling pathway were identified in our transcriptome data. The genes involved in the SA signaling were upregulated in the reproductive stage. While the genes from other hormone signaling pathways have no obvious expression trend (Additional file 18: Fig. S8b).

### Identification of floral transition- and flower development-related genes

**Fig. 5:**
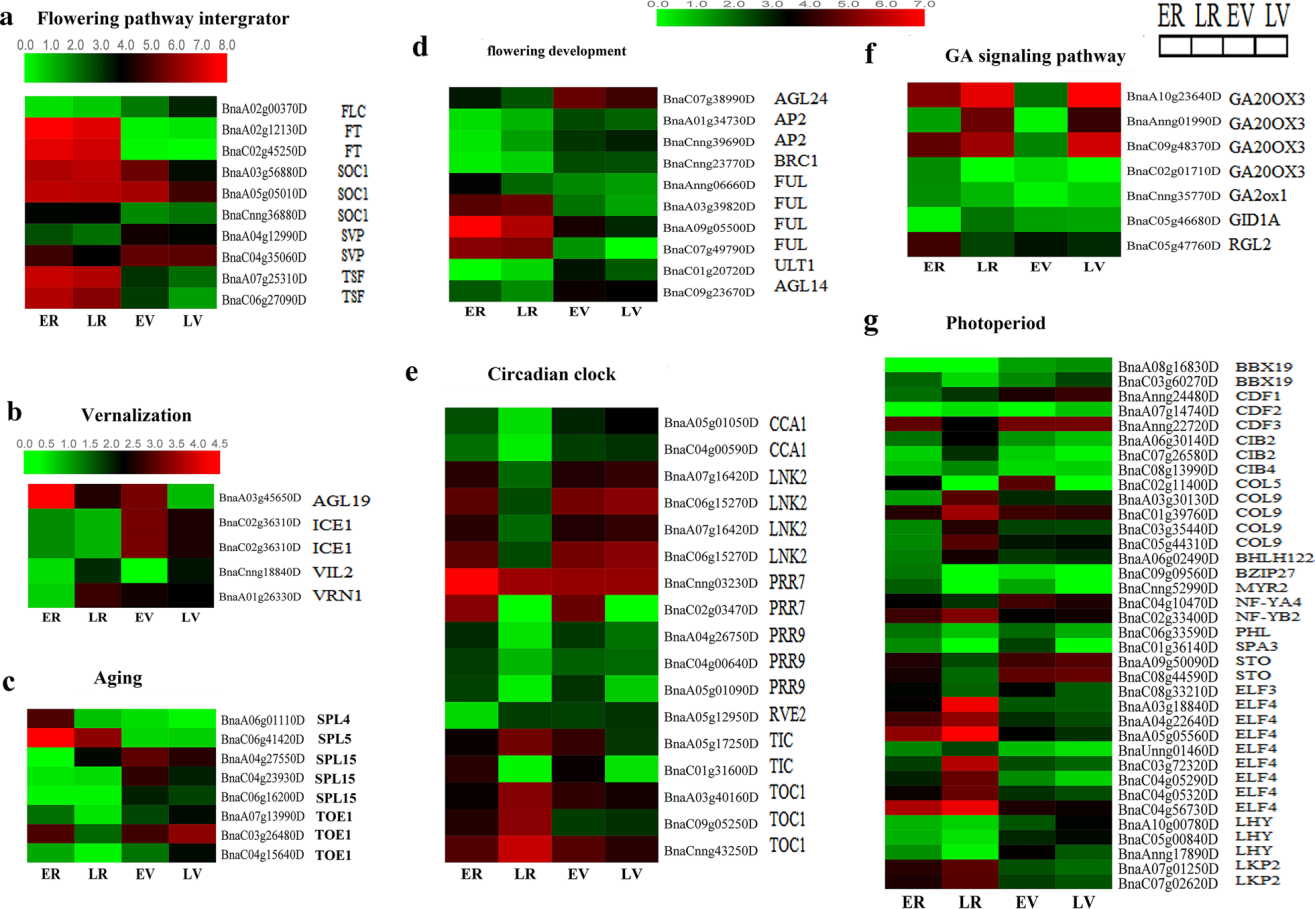
Heatmap showing expression patterns of DEGs involved in the flowering development including flowering pathway integrator (**a**), vernalization pathway (**b**), aging pathway (**c**), non-classified flowering regulators (**d**), circadian clock (**e**), GA signaling pathway (**f**) and photoperiod pathway (**g**). Gene expression levels were transformed with log2 (FPKM + 1)

According to the annotation of unigenes in *Arabidopsis*, a total of 125 DEGs related to flowering time were identified in the comparisons LR vs. ER, LV vs. EV, EV vs. ER and LV vs. LR (Additional file 19: Table S11). These genes mainly included the photoperiod, circadian rhythms, vernalization, GA signaling, aging, flowering time integrator and flower meristem identity genes, and the expression value of these genes in the four samples is shown in Fig. 5.

In the photoperiod pathway, 36 unigenes homologous to *EARLY FLOWERING 3* (*ELF3*), *EARLY FLOWERING 4* (*ELF4*), *CONSTANS*-*like 9* (*COL9*), *cycling DOF factor 1* (*CDF1*) and *LATE ELONGATED HYPOCOTYL* (*LHY*) were identified. The circadian rhythm, as an internal timekeeper, controls daily and seasonal changes, is an important part of the photoperiod pathway and plays a key role in controlling plant flowering [29]. *Circadian clock associated 1* (*CCA1*) is a key component of the *Arabidopsis* circadian oscillator, and it interacts with *LATE ELONGATED HYPOCOTYL* (*LHY*) and *TIMING OF CAB EXPRESSION 1* (*TOC1*) to inhibit transcription of the Evening Complex (EC) proteins ELF4 and ELF3. In the circadian clock pathway, two *CCA1* genes, three *TOC1* genes, two *pseudo response regulator 7* (*PRR7*) genes and three *pseudo response regulator 9* (*PRR9*) genes were included. For the aging pathway, eight unigenes, including *SQUAMOSA PROMOTER*-*BINDING*-*LIKE PROTEIN 4* (*SPL4*), *SQUAMOSA PROMOTER*-*BINDING*-*LIKE PROTEIN 5* (*SPL5*), *SQUAMOSA PROMOTER*-*BINDING*-*LIKE PROTEIN 15* (*SPL15*) and *TARGET OF EARLY ACTIVATION TAGGED 1*(*TOE1*) were found. Seven homologous genes of the GA signaling pathway were also identified, including *GA2 oxidase* (*GA2ox*, three unigenes), *GA2 oxidase 1* (*GA2ox1*), *gibberellin 20*-*oxidase 3* (*GA20OX3*), *DELLA protein RGA*-*like 2* (*RGL2*), and the GA receptor *GA INSENSITIVE DWARF1A* (*GID1A*). Additionally, five unigenes were annotated in the vernalization pathway, which included the *AGAMOUS*-*like 19* (*AGL19*), *INDUCER OF CBF EXPRESSION 1* (*ICE1*), *vernalization5/VIN3*-*like* (*VEL1*) and *REDUCED VERNALIZATION RESPONSE 1* (*VRN1*). Furthermore, ten floral pathway integrator genes related to *FLC*, *FLOWERING LOCUS T* (*FT*), *AGAMOUS*-*like 20* (*AGL20*), S*HORT VEGETATIVE PHASE* (*SVP*) and *TWIN SISTER OF FT* (*TSF*) and ten flowering meristem-identifying genes, such as *AGAMOUS*-*like 8* (*AGL8*), *AGAMOUS*-*like 14* (*AGL14*), *AGAMOUS*-*like 24* (*AGL24*) and *APETALA 2* (*AP2*), were all identified in our transcriptome database. All these DEGs are important resources for the further study of floral transition and floral development in *B. napus*.

### Identification of candidates for growth period traits by integrating QTLs with DEGs

**Fig. 6:**
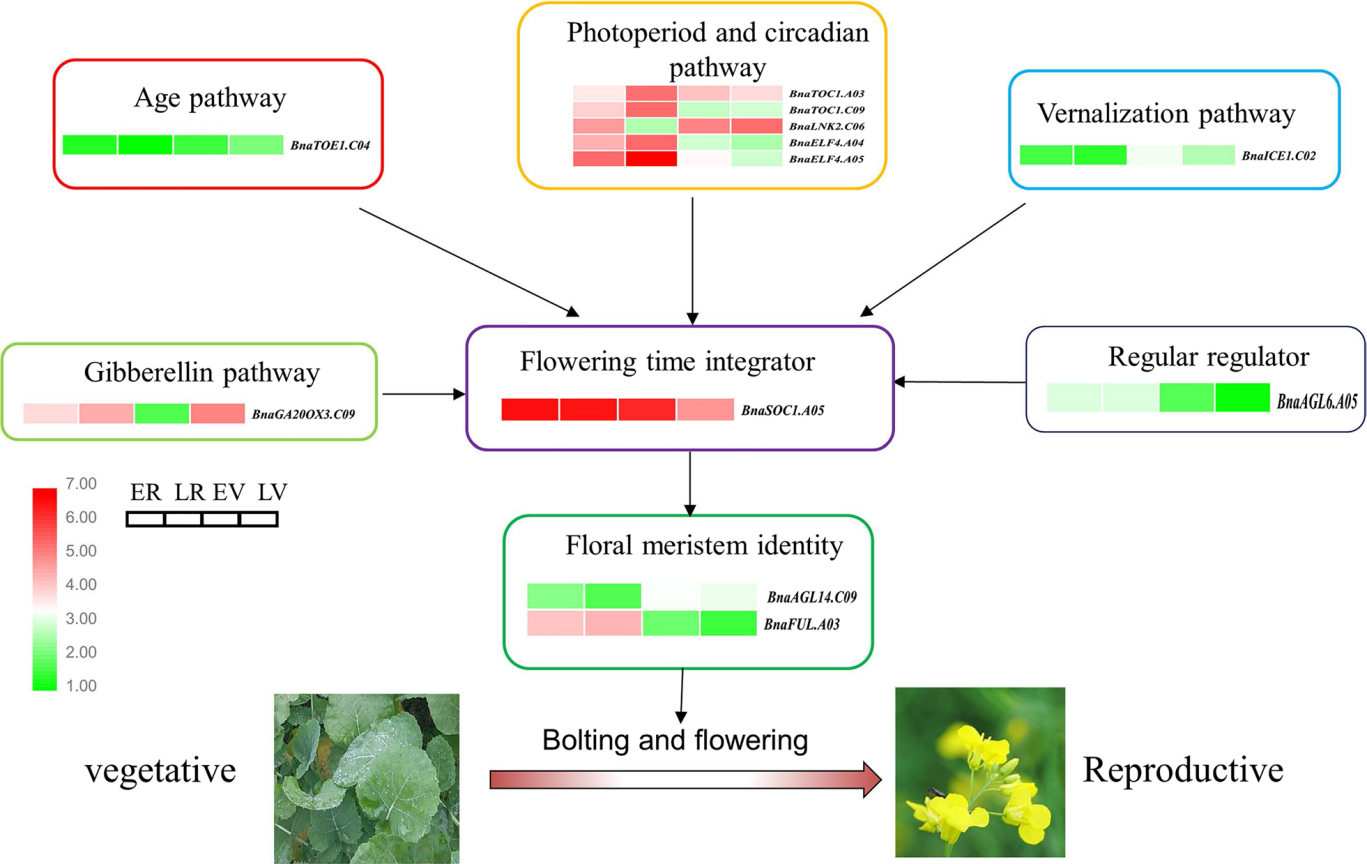
The putative model of flowering regulatory network of 12 candidate genes associated with flowering time in *B. napus*. The heatmap represents the expression levels in four samples

To further understand the roles of these DEGs in regulating floral transition and flower development, the DEGs were integrated with the significant QTLs identified in either association analysis or linkage mapping. Therefore, the DEGs were considered candidate genes if they were located within the confidence interval (CI) of the QTLs identified by GWAS, linkage mapping, or both. According to the above criteria, a total of 12 DEGs located in the CI of 16 significant loci were identified as candidate genes of growth period traits. The loci were *BnaC04g15640D*, *BnaA03g40160D*, *BnaC06g15270D*, *BnaC09g05250D*, *BnaA04g22640D*, *BnaA05g05560D*, *BnaA05g05000D*, *BnaC09g48370D*, *BnaC02g36310D*, *BnaC09g23670D*, *BnaA03g39820D* and *BnaA05g05010D*. The 12 DEGs were known to regulate floral development by affecting aging, photoperiod/circadian clock, GA, vernalization, flower development and meristem identity and flowering time integrator (Table 4 and Fig. 6). *BnaAGL6.A05*, which encodes a MADS-box transcription factor, negatively regulates the *FLC/MAF* clade genes and positively regulates *FT* in *Arabidopsis* [30]. Among them, *BnaC06g15270D* (*BnaLNK2.C06*), *BnaC09g48370D* (*BnaGA20OX3.C09*) and *BnaA03g39820D* (*BnaFUL.A03*) were also identified as EDGs between BBCH20 and BBCH50 (vernalized and nonvernalized) associated with flowering time and yield QTLs [27]. In addition, an ortholog of the circadian clock pathway, *BnaTOC1.A03* and floral meristem identity gene *BnaFUL.A03* were identified in both GWAS and linkage analysis [31]. Thus, we considered these five genes to be our most promising candidate genes for future prospects.

**Table 4:** Candidate genes identified by integrating RNA-seq with linkage mapping or GWAS

*B. napus* gene ID	Ath homolog	Gene name	Gene annotation	GWAS QTL	Linkage mapping QTL	log2(ER/EV)	log2(ER/LR)	log2(EV/LV)	log2(LR/LV)
*BnaC04g15640D*	*TOE1*	*BnaTOE1.C04*	TARGET OF EARLY ACTIVATION TAGGED 1	S14_13322100, S14_13322121	–	–	–	− 1.22	–
*BnaA03g40160D*	*TOC1*	*BnaTOC1.A03*	TIMING OF CAB EXPRESSION 1	S3_20114553	q18MT.A03-2	–	− 1.78	–	1.56
*BnaC09g05250D*	*TOC1*	*BnaTOC1.C09*	TIMING OF CAB EXPRESSION 1	S19_30931596	–	1.45	− 1.46	–	2.71
*BnaC06g15270D*	*LNK2*	*BnaLNK2.C06*	NIGHT LIGHT-INDUCIBLE AND CLOCK-REGULATED 2	S16_17975794	–	–	2.47	–	− 3.12
*BnaA04g22640D*	*ELF4*	*BnaELF4.A04*	EARLY FLOWERING 4	–	q18FP.A04	1.73	− 1.04	–	3.24
*BnaA05g05560D*	*ELF4*	*BnaELF4.A05*	EARLY FLOWERING 4	S5_3021806	–	2.10	− 1.49	–	4.29
*BnaA05g05000D*	*AGL6*	*BnaAGL6.A05*	AGAMOUS-LIKE 6	–	q17DFF.A05-2	1.99	–	–	–
*BnaC09g48370D*	*GA20OX3*	*BnaGA20OX3.C09*	GIBBERELLIN 20-OXIDASE 3	S19_47135806	–	3.12	–	− 4.40	–
*BnaC02g36310D*	*ICE1*	*BnaICE1.C02*	INDUCER OF CBF EXPRESSION 1	S12_39314951	–	− 2.62	–	–	–
*BnaC09g23670D*	*AGL14*	*BnaAGL14.C09*	AGAMOUS-LIKE 14	–	q17GP.C09	− 1.48	–	–	− 2.27
*BnaA03g39820D*	*FUL*	*BnaFUL.A03*	FRUITFULL	S3_20114553	q18MT.A03-2	2.89	–	–	4.10
*BnaA05g05010D*	*SOC1*	*BnaSOC1.A05*	SUPPRESSOR OF OVEREXPRESSION OF CO 1	–	q17DFF.A05-2	–	–	1.56	1.83

To analyze the polymorphism and explore their relationship with five growth traits, the sequence variation was analyzed in the genomic sequence including the complete coding sequence and 2000 bp upstream of the ATG translational start codon in the 12 candidate genes in the 558 lines based on our resequencing data. Among 12 genes, seven genes had polymorphic sites: 1, 15, 4, 5, 1, 3 and 10 SNPs were identified in the *BnaFUL.A03*, *BnaELF4.A04*, *BnaSOC1.A05*, *BnaELF4.A05*, *BnaLNK2.C06*, *BnaTOC1.C09* and *BnaGA20OX3.C09,* respectively, and formed different types of haplotypes. Detailed haplotype information is listed in Additional file 20: Table S12.

The association of each haplotype with five growth periods was then analyzed in the GWAS population. We compared the phenotypic variations of different haplotypes for the above agronomic traits (Additional file 20: Table S12, Additional file 21: Fig. S9 and Additional file 22: Fig. S10). In general, for *BnaELF.A04*, accessions with BnaELF.A04-Haplc accounted for 94.2% and showed a significantly shorter MT period in 2018 and 2019 compared to BnaELF.A04-Hapla. For *BnaELF.A05,* varieties with BnaELF.A05-Hapla accounted for 90.8% and showed the shortest FP compared to the other two haplotypes in 2017 and 2019. For *BnaSOC1.A05*, most accessions have BnaSOC1.A05-Haplb (97.5%) and exhibited shorter DFF and longer MT over three years compared to the BnaSOC1.A05-Hapla. For *BnaKNK2.C06*, most varieties have BnaKNK2.C06-Hapla (93.7%) and showed lower DFF and GP than BnaKNK2.C06-Haplb. For *BnaTOC1.C09*, accessions with BnaTOC1.C09-Haplc accounted for 94.1% and had lower DFF, lower FP and longer MT over 3 years. For *BnaGA20OX3.C09*, the two haplotypes did not exhibit significant differences between the five growth period traits. Thus, the haplotype BnaSOC1.A05-Haplb and BnaLNK2.C06-Hapla showed more favorable phenotypic traits and may be used for further earliness molecular breeding.

### Confirmation of candidate gene expression using qRT- PCR

**Fig. 7:**
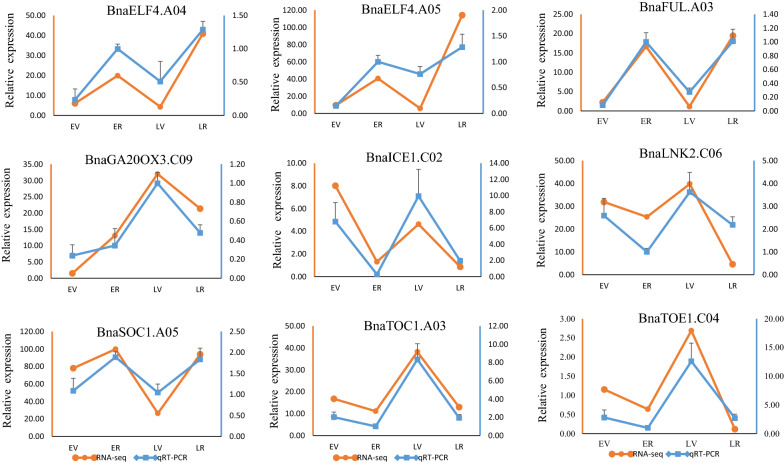
qRT-PCR validation of the expression patterns of nine candidate genes in four samples. The orange line represents the RNA-Seq results, the blue line represents the qRT-PCR results and data are shown as mean ± SEM

To verify the accuracy and reproducibility of the transcriptome analysis, nine candidate DEGS listed in Table 4 were selected for qRT-PCR analysis. As shown in Fig. 7, the expression of nine genes were consistent with the RNA-Seq results in the four samples. These results demonstrated the reliability of the RNA-sequencing results.

## Discussion

Growth period are important traits that can influence crop yield and quality. Identifying the genetic loci for growth period traits may help to elucidate the genetic basis underlying the growth period and be valuable for developing cultivars adapted to different geographical regions. Linkage analysis and association mapping are the two most common methods used for mapping complex traits. Linkage mapping can exclude false positives of associated loci caused by high linkage disequilibrium, and association mapping can be used to narrow the confidence interval of linkage analysis when its QTL regions are large. The combination of two mapping methods further improves mapping efficiency and accuracy [32–34]. In the current study, we detected the SNP loci associated with five growth period traits (DIF, DFF, FP, MT, GP) via GWAS in the natural population in three-year environments and detected QTLs that influence growth in a RIL population over 2 years in *B. napus*.

A total of 146 SNP loci were found to be associated with growth period traits (13 related to DIF, 23 to DFF, 6 to FP, 19 to MT and 85 to GP), which were detected in at least two environments (including BLUP). Among these SNP loci, 28 were consistent with at least one QTL identified in one or more previous studies. In addition, 101 candidate genes were identified as flowering orthologous genes based on the LD block or 300 kb to either side of the significant SNPs, most of which were also reported in previous studies [14, 22–28]. In particular, the flowering time integrator and floral meristem identify genes (*BnaFLC.C2*, *BnaFUL.A3*, and *BnaSVP.A9*) were reported in at least three studies. These results indicated that the results in the present study are notably reliable. A total of 83 QTLs were observed relating to DIF, DFF, FP, MT and GP by linkage mapping in 2 years. These QTLs could explain over 10% of the phenotypic variance for DIF, DFF, FP, MT and GP. However, most QTLs were only detected in a single-year environment, which indicates that the rapeseed growth period was largely affected by environmental variation. Finally, six QTLs were associated with growth period traits using both association and linkage mapping (Additional file 23: Table S13). S19_17595451 and q17GP.C09 were associated with GP. However, the other five QTLs with overlapping confidence intervals were related to different traits in linkage analysis and association analysis. The results were also reported in Zuo et al. [35].

### SNP markers with pleiotropic effects

Some GWAS and linkage mapping studies have indicated that one locus can control multiple highly correlated traits [33, 36]. We detected significant correlations between the five growth period traits (Table 2), therefore, it is possible that some SNPs can affect these traits simultaneously. Five QTLs found in the GWAS and 15 QTLs identified in the linkage mapping were co-associated with at least two growth period traits, which coincided with significant phenotypic correlations among the traits (Additional file 24: Table S14a, b). It has been reported that co-mapping of QTLs for correlated traits may result from either tight linkage of multiple genes [37] or single gene pleiotropy [38]. In our research, we found that a single gene can control multiple traits (Additional file 25: Fig. S11). This finding further confirmed that co-mapping of QTLs caused by single gene pleiotropy. The co-mapping of QTLs can help breeders identifying favorable alleles for multiple traits simultaneously in marker-assisted breeding [39].

### Identification of flowering DEGs

A GO and KEGG pathway enrichment analysis indicated that the common DEGs between EV vs. ER and LV vs. LR were enriched for circadian rhythm—plant and salicylic acid biosynthetic process (Additional file 15: Fig. S6b), indicating that circadian rhythm and salicylic acid play an important role in the *B. napus* floral transition, which was also reported in the *Annona squamosa* and *Rosa chinensis* [40, 41]. We also observed significant enrichment for genes involved in the circadian rhythm between LR vs. ER, suggesting that the flowering time difference between early- and late-flowering lines was primarily due to the expression difference of circadian rhythm genes (Additional file 17: Fig. S7b, d).

Transcription factors have been reported to play crucial roles in the reproductive development of flowering plants [42]. The MADS-box transcription factor is a major group of regulators controlling floral transition, floral organ specification and floral development in flowering plants [43].In the present study, the bHLH, ERF, MIKC_MADS, NAC and WRKY transcription factors were identified. Most IF genes were upregulated during the floral transition of *B. napus* (Additional file 18: Fig. S8a), suggesting their pivotal roles in the induction of floral transition. Plant hormones, such as auxin, cytokinin (CK), abscisic acid (ABA), gibberellic acid (GA), salicylic acid (SA), and jasmonic acid (JA), are involved in the floral transition process and plant flowering [44–46]. The effects of GA on flowering have been extensively studied in *Arabidopsis* [47, 48]. 62 DEGs involved in six hormone pathways were identified in our transcriptome data, which further confirmed the importance of hormones in the flowering and flower development process (Additional file 18: Fig. S8b).

In this study, we also detected 125 flowering and flower development-related homologous genes based on BLAST analysis using sequences of 306 known flowering genes in *Arabidopsis*. Some of these genes are related to five major flowering pathways, including circadian rhythms/photoperiod, vernalization, GA signaling and aging, while others encode regulators functioning as flowering integrators or floral meristem identification (Fig. 5 and Additional file 19: Table S11) [49].

### Candidate genes for growth period traits

The integration of QTL mapping and transcriptome sequencing is a highly useful and popular strategy for discovering candidate genes of complex traits [18]. By integrating DEGs from RNA-seq with GWAS and linkage mapping, 12 genes mainly related to circadian clock, hormone, flowering time integrator and flower meristem identity were identified as candidate genes regulating growth period traits in *B. napus*.

Of the 12 candidate genes detected in this study, five genes are involved in the circadian clock/photoperiod pathways: *BnaA03g40160D* and *BnaC09g05250D* are homologous to *TOC1* and interact with *LHY,* and *CCA1* forms the negative feedback loop of the circadian clock [29]; *BnaA04g22640D* and *BnaA05g05560D* are homologous to *EARLY FLOWERING 4* (*ELF4*), which is a part of a corepressor complex consisting of *ELF4*, *ELF3*, and *LUX* involved in the transcriptional regulation of *APRR9* and *elf4* mutations result in early flowering in non-inductive photoperiods [50]; *BnaC06g15270D* is homologous to *NIGHT LIGHT*-*INDUCIBLE AND CLOCK*-*REGULATED 2* (*LNK2*), *LNK1* and *LNK2* integrate early light signals with core oscillator components (*TOC1*, *PRR*) to keep track of seasonal changes in day length [51]. The up-regulation of *TOC1* and *ELF4* in the late-flowering lines (LR) was in accordance with the mutant phenotype in *Arabidopsis*; *BnaC02g36310D* homologous to *ICE1* is involved in vernalization pathways. Cold‐activated *ICE1* directly induces the expression of *FLC*, which represses *SOC1* expression, resulting in delayed flowering [52]; *BnaC09g48370D* is homologous to *GA20OX3*, which encodes a gibberellin 20-oxidase involved in the GA pathway. Both *GA20ox* and *GA* *2*-*oxidase* genes are regulated by several transcription factors, including *SHORT VEGETATIVE PHASE* (*SVP*) and *SOC1* [53–55]; *BnaC04g15640D* encodes a gene homologous to *TOE1*, which is a type of *AP2* transcription factor and the target gene of miR172. *TOE1* and its microRNA regulator miR172 play an important role in plant growth and phase change in different crops [56–58]; *BnaA05g05000D* is homologous to *AGL6* (encoding a MADS-box transcription factor). *AGL6* regulates flowering time through control the transcription of two key regulators of flowering time: *FLOWERING LOCUS C* (*FLC*) and *FT*. The *agl6*-*1D* mutant, in which *AGL6* was activated by the 35S enhancer, showed early flowering under both long-day (LD) and short-day (SD) conditions [59]; the floral integrator genes play important roles in activating floral meristem formation genes to induce flowering. *BnaA05g05010D* was identified as a *SOC1* paralog, which is a floral activator and integrates signals from the photoperiod, vernalization, and autonomous pathways in *Arabidopsis* [60, 61]. Overexpression of *SOC1* could suppress the late flowering and delayed phase transitions during the vegetative stages [62]; *BnaA03g39820D* was homologous to *FRUITFULL* (*FUL*) of *Arabidopsis*, a MADS-box transcription factor negatively regulated by *APETALA1*(*AP1*), which mediates the vegetative and meristem identity transitions by forming *FUL*-*SVP* and *FUL*-*SOC1* heterodimers. *FUL* has been reported to connect several flowering pathways as a downstream target gene of age, photoperiod, and ambient temperature pathways [31]; *BnaC09g23670D* is homologous to *AGL14*, *AGL14* promote flowering transition and participate in flower meristem maintenance and determinacy by positively regulating *TERMINAL FLOWER 1* (*TFL1*) expression [63, 64].

## Conclusions

In this context, we conducted systematic research (including GWAS, linkage mapping and gene expression analysis) to identify candidate genes regulating growth period traits in *B. napus*. In summary, 146 SNPs and 83 QTLs associated with the five growth period traits (DIF, DFF, FP, MT and GP) were identified by association and linkage mapping techniques. Six QTLs were associated with growth period traits using both mapping methods. Five QTLs found in the GWAS, and 14 QTLs identified in the linkage mapping were identified as pleiotropic SNPs. RNA-Seq analysis demonstrated that the genes mediated by photoperiod/circadian rhythms, vernalization, GA signaling, aging, flowering time integrator and flower meristem identity may regulate floral transition and flower development in *B. napus*. An integrated analysis of QTLs and DEGs identified 12 candidate genes for growth period traits. *BnaTOC1.A03* and *BnaFUL.A03* were identified in both GWAS and linkage analysis; *BnaLNK2.C06*, *BnaGA20OX3.C09* and *BnaFUL.A03* have been previously reported. Among 12 genes, 7 genes have polymorphic sites in the coding sequence and 2000 bp upstream of the TSS. According to phenotype analysis, the haplotype BnaSOC1.A05-Haplb and BnaLNK2.C06-Hapla showed more favorable phenotypic traits. Therefore, our data suggest that these genes may play important roles as regulators of flowering time and growth period in rapeseed.

## Materials and methods

### Plant material and phenotypic evaluation

A diversity panel consisting of 588 rapeseed inbred lines (74 winter types, 428 semi-winter types, and 86 spring types) was used for the GWAS analysis in the present study [24]. These lines were grown at the Southwest University of Beibei (29°45′N latitude, 106°22′E longitude, and an altitude of 238.57 m), Chongqing, China, in 2017, 2018 and 2019. Each variety was planted in a plot with two rows (30-cm line width and 20-cm plant distance), and each row had 10 plants. A randomized complete block design with two replications was employed.

The RIL populations of 172 RILs derived from GH06 × P174 were previously described in Liu et al. [65]. The genetic linkage map contains 9164 SNP markers covering 1832.9 cM, and 2795 SNPs were applied for QTL mapping. The population was planted in the experimental field of Southwest University of Beibei (29°45′N latitude, 106°22′E longitude, and an altitude of 238.57 m) Chongqing, China, in 2017 and 2018. The planting method is consistent with the GWAS population.

Five growth period traits, DIF (the number of days from sowing to the date when 25% plants opened first flower), DFF (the number of days from sowing to the date when 75% plants had stopped blooming), FP (the difference between DFF and DIF), MT (the number of days from DFF to the date when 75% pods were yellow) and GP (the number of days from sowing to the pod ripen) were recorded in the field trials, respectively [14]. Flowering was scored in winter, semi-winter and spring types using the same standard [15, 24, 28]. Analysis of variance (ANOVA) was performed using the GLM procedure of SAS.

### Genome-wide association analysis

GWAS for the growth period traits were performed using Tassel 5.0 software by general linear models (GLM) and mixed linear models (MLM) methods. Population structure, relative kinship, and LD analysis were completed in previous studies [24]. A negative log (1/n) was used as a threshold for significant association SNPs with traits, where n represents the SNP number used in the GWAS [66, 67]. Significant markers in the same LD block were viewed as one QTL region. The QQ plot and Manhattan plot were displayed using qqman [68] and CMplot software.

### QTL analysis

Windows QTL Cartographer Version 2.5 (WinQTLcart2.5) software was used in QTL mapping via the composite interval mapping method [69]. The logarithm of the odds (LOD) threshold for a significant QTL was calculated with 1000 permutations at a significance level of *p* = 0.05. A confidence interval for each QTL was defined by LOD change from the peak position. The contribution rate (R^2^) and additive effect of a putative QTL were also calculated from WinQTLcart2.5. If the phenotypic variance explained was larger than 10%, we classified the QTL as a major QTL. QTLs were assigned names by adding the prefix of trait abbreviation, and their locations, including the chromosome number, were determined according to McCouch et al. [70]. If more than one QTL was detected on the same chromosome for a trait, QTLs were serially numbered. MapChart software was used to draw the QTL position [71].

### Gene expression analysis

Early-flowering material (18Z134) and late-flowering material (18Z88) were selected from the above RIL population based on 6 years of field data. The juvenile leaves at the vegetative stage (30 days after seeding when six leaves fully expanded) and reproductive stage (20 days after flowering when 50% of the flowers are open) were collected, respectively, and immediately placed in liquid nitrogen and stored at − 80 °C. Total RNA was extracted using RNAprep Pure Tissue Kit (Tiangen, China) according to the manufacturer’s instructions. RNA was pooled to yield four RNA samples, EV (leaves of early-flowering material at vegetative stage), LV (leaves of late-flowering material at vegetative stage), ER (leaves of early-flowering material at reproductive stage) and LR (leaves of late-flowering material at reproductive stage). Each sample has two biological replications. The sequencing data have been deposited in NCBI Sequence Read Archive (SRA, https://www.ncbi.nlm.nih.gov/sra) with the accession number PRJNA540020.

DEGs between the two samples were identified based on the criteria false discovery rate (FDR) < 0.05 and |log2 (Fold change) | > 1. Gene ontology (GO) enrichment analysis of the DEGs was performed using Blast2GO, and the significantly enriched GO terms (*p* < 0.05) were displayed using the online tool WEGO (http://wego.genomics.org.cn). Kyoto Encyclopedia of Genes and Genomes (KEGG) pathway enrichment analysis of the DEGs was performed using the KOBAS2.0 website (http://kobas.cbi.pku.edu.cn/home.do). The transcript abundance was calculated based on the fragments per kilobase of exon model per million mapped reads (FPKM). The log2-transformed (FPKM + 1) values were used to study gene expression.

### Candidate gene predication

According to the decay of LD, genes within 300 kb up and downstream of the significantly associated SNP loci were mined [12, 72]. The QTL intervals were aligned to the *B. napus* reference genomes (http://www.genoscope.cns.fr/blat-server/cgi-bin/colza/webBlat). Based on the physical positions of the flanking marker, genes located in the QTL confidence interval were extracted. The prediction of candidate genes referred to the two conditions: genes of known function in *B. napus* or genes with function-known orthologs in *Arabidopsis.* Genes within the confidence interval and meeting one of the above conditions were used for gene expression analysis.

### Validation of candidate genes by qRT–PCR

For qRT-PCR, the samples used for RNA-seq were used to synthesize cDNA using the QuantiTect^®^ Reverse Transcription Kit (Qiagen), and qRT–PCR experiments were performed with SYBR qPCR Mix (Bio-Rad) according to the manufacturer’s specification. At least three biological replicates along with two technical replicates were analyzed for expression levels. BnActin7 (5′-GGAGCTGAGAGATTCCGTTG-3′ and 5′-GAACCACCACTGAGGACGAT-3′) was used as an internal control. The 2^−ΔΔCt^ method was used to calculate the normalized expression of target genes [73]. The candidate gene-specific primers are shown in Additional file 26: Table S15.

## Supplementary information

**Additional file 1: Table S1.** ANOVA analysis of five growth period traits in GWAS(a) and linkage mapping(b).

**Additional file 2: Fig. S1.** SNP density map of GWAS.

**Additional file 3: Fig. S2.** The QQ plots from the GWAS of DIF, DFF, FP, MT and GP using BLUP value. DIF, days to initial flowering; DFF, days to final flowering; FP, flowering period; MT, maturity time; GP, growth period.

**Additional file 4: Table S2.** List of significant SNPs repeatedly detected for growth period traits by GWAS in *B. napus*.

**Additional file 5: Table S3.** LD block analysis and SNP/candidate genes comparison in different studies.

**Additional file 6: Table S4.** Genes identified in LD block or 300 kb up- and down-stream of significant SNPs identified in GWAS.

**Additional file 7: Fig. S3.** Histogram of the frequency distribution of five growth period traits in RIL population in 2017 and 2018.

**Additional file 8: Fig. S4.** The LOD score plots of five growth period traits across linkage groups in 2017(a) and 2018(b). Different traits are indicated by lines with various backgrounds (Red: QTL for initial flowering; green: the final flowering; blue: flowering period; black: maturity time; yellowish green: growth period).

**Additional file 9: Table S5.** QTLs detected for five growth period traits in GH06 × P174 RIL population.

**Additional file 10: Table S6.** Genes located in the confidence interval of significant QTLs identified by linkage mapping.

**Additional file 11: Table S7.** The differences of flowering time between early- and late- flowering *B. napus* lines.

**Additional file 12: Table S8.** Summary of read numbers and mapped reads from the RNA-Seq of four samples (EV, ER, LV and LR). EV: leaves of early-flowering at vegetative stage, LV: leaves of late-flowering at vegetative stage, ER: leaves of late-flowering at reproductive stage, LR: leaves of late-flowering at reproductive stage.

**Additional file 13: Fig. S5.** The statistics of differential expression gene in four comparisons. a, the Venn diagram of DEGS identified in LR vs. ER and LV vs. EV; b, the Venn diagram of DEGS identified in EV vs. ER and LV vs. LR; c, the number of up-regulated and down-regulated genes identified in the comparisons of four groups.

**Additional file 14: Table S9.** The DEGs number between four samples using FDR < 0.05 and |log2 (Fold change) | > 1 as the significant cutoff.

**Additional file 15: Fig. S6.** Go and KEGG pathway significantly overrepresented of common genes between EV vs. ER and LV vs. LR. a, the enriched GO terms; b, the top 20 enriched pathways.

**Additional file 16: Table S10.** Significantly overrepresented GO terms (a) and KEGG pathways (b) in the common genes of EV vs. ER and LV vs. LR.

**Additional file 17: Fig. S7.** Go terms and KEGG pathways enriched between the two extreme lines at each development stage. a, the enriched GO terms in LV vs. EV; b, the enriched GO terms in LR vs. ER; c, the top 20 pathways enriched in LV vs. EV; d, the top 20 pathways enriched in LR vs. ER.

**Additional file 18: Fig. S8.** Heatmap diagram of expression levels for DEGs involved in floral transition and flowering development-associated transcription factors (a) and phytohormone signaling pathways including ABA, auxin, JA, GA and SA (b).

**Additional file 19: Table S11.** List of flowering time genes in *B. napus* from our RNA-sequencing data using *Arabidopsis* flowering genes as queries.

**Additional file 20: Table S12.** The detailed information of different haplotypes detected in seven candidate genes.

**Additional file 21: Fig. S9** The box-plot of different haplotypes of *BnaFUL.A03*, *BnaELF4.A04* and *BnaELF4.A05* on the basis of five growth periods in three years.

**Additional file 22: Fig. S10** The box-plot of different haplotypes of *BnaSOC1.A05*, *BnaLNK2.C06*, *BnaTOC1.C09* and *BnaGA20OX3.C09* on the basis of five growth periods in three years.

**Additional file 23: Table S13.** The common QTLs detected by both GWAS and linkage mapping.

**Additional file 24: Table S14.** List of SNP markers with pleiotropic effect.

**Additional file 25: Fig. S11.** The venn diagram of the number of flowering genes with pleiotropic effect identified in GWAS and linkage mapping.

**Additional file 26: Table S15.** Gene-specific primers used for qRT-PCR verification. *Bnactin7* is the internal reference gene.

## Data Availability

All experimental materials are available on request. All data generated during this study are included in this article and its additional files.

## Abbreviations

DIF: Days to initial flowering
DFF: Days to final flowering
FP: Flowering period
MT: Maturity time
GP: Growth period
GWAS: Genome-wide association study
GLM: General linear model
MLM: Mixed linear model
SNP: Single nucleotide polymorphism
MAF: Minor allele frequency
QTL: Quantitative trait locu
BLUP: Best linear unbiased prediction
EV: Leaves of early-flowering material at vegetative stage
LV: Leaves of late-flowering material at vegetative stage
ER: Leaves of early-flowering material at reproductive stage
LR: Leaves of late-flowering material at reproductive stage
RNA-seq: RNA sequencing
DEGs: Differentially expressed genes
FPKM: Fragments per kilobase of exon per million reads mapped
FDR: False discovery rate
qRT-PCR: Quantitative real-time PCR
TOE1: Target of early activation tagged 1
TOC1: Timing of cab expression 1
LNK2: Night light-inducible and clock regulated 2
ELF4: Early flowering 4
AGL6: AGAMOUS-like 6
GA20OX3: Gibberellin 20-oxidase 3
ICE1: Inducer of CBF expression 1
AGL14: AGAMOUS-Like14
FUL: FRUITFULL
SOC1: Suppressor of overexpression of CO 1
