# Supplementary figures and images for: Integrating GWAS, linkage mapping and gene expression analyses reveals the genetic control of growth period traits in rapeseed (Brassica napus L.)
Source: Biotechnol Biofuels. 2020 Aug 3;13:134. doi: 10.1186/s13068-020-01774-0 (PMC7397576; doi:10.1186/s13068-020-01774-0)

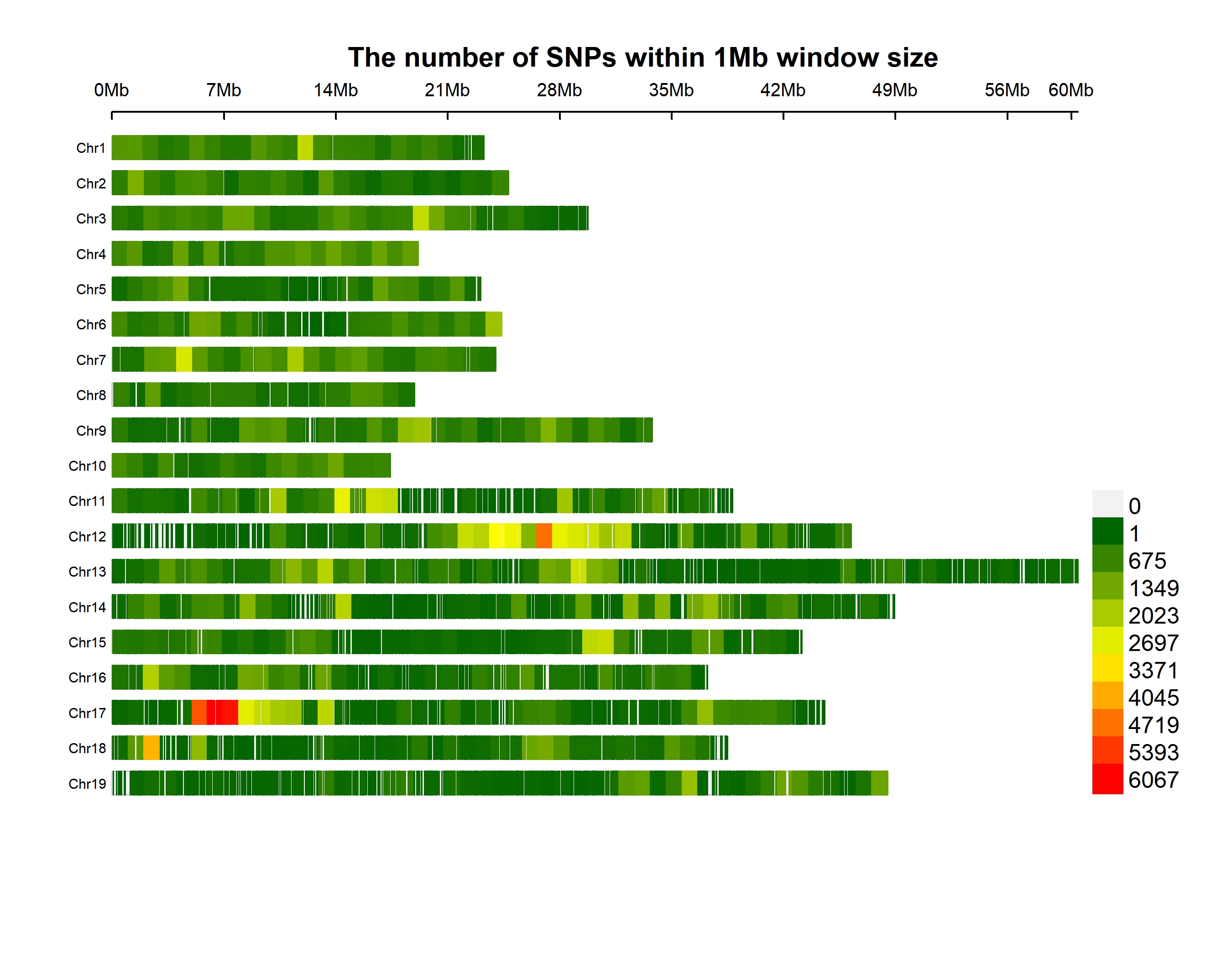

Supplement: Supplementary file 2 — Additional file 2: Fig. S1. SNP density map of GWAS. [file 13068_2020_1774_MOESM2_ESM.jpg]

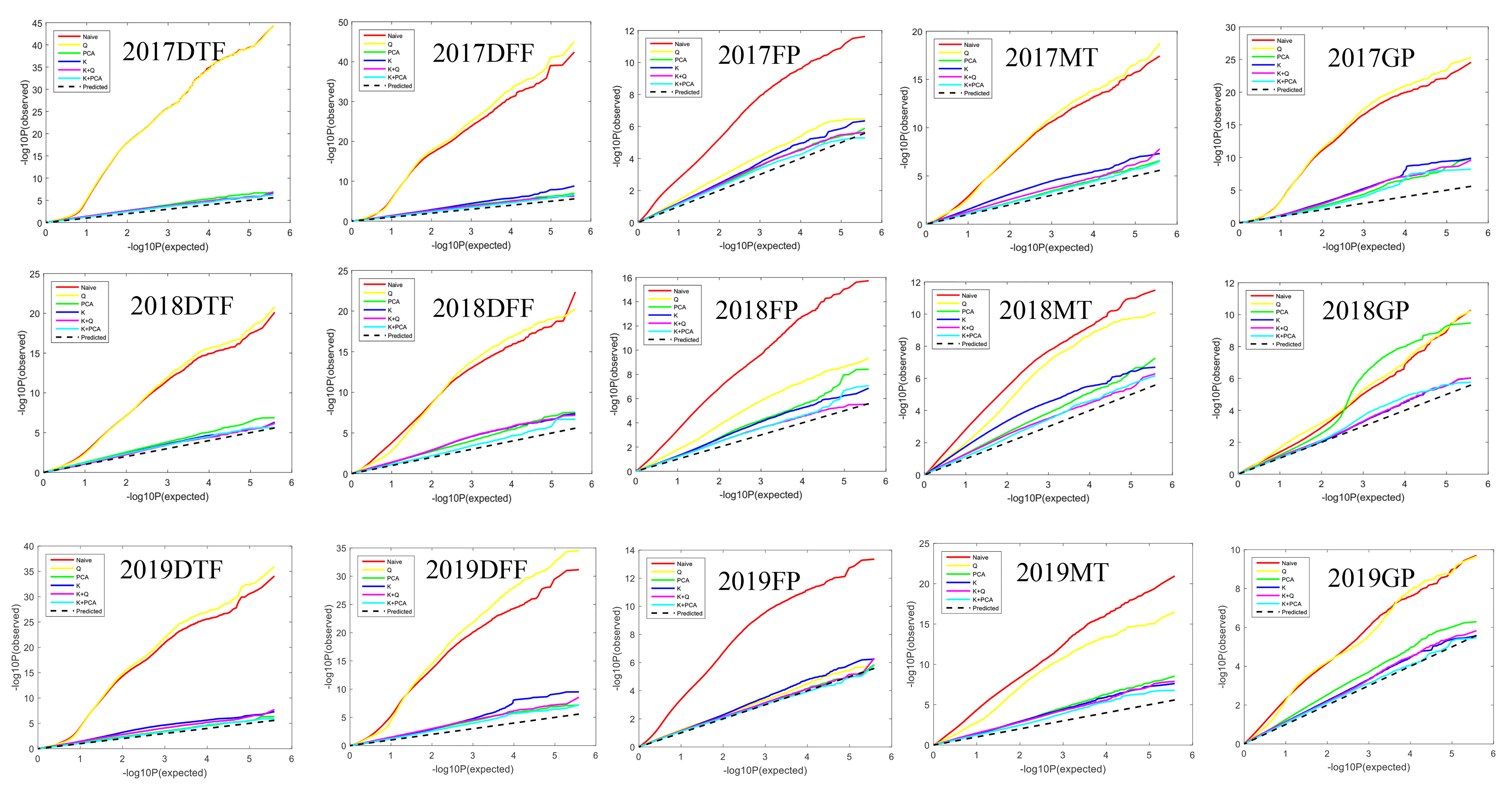

Supplement: Supplementary file 3 — Additional file 3: Fig. S2. The QQ plots from the GWAS of DIF, DFF, FP, MT and GP using BLUP value. DIF, days to initial flowering; DFF, days to final flowering; FP, flowering period; MT, maturity time; GP, growth period. [file 13068_2020_1774_MOESM3_ESM.tif]

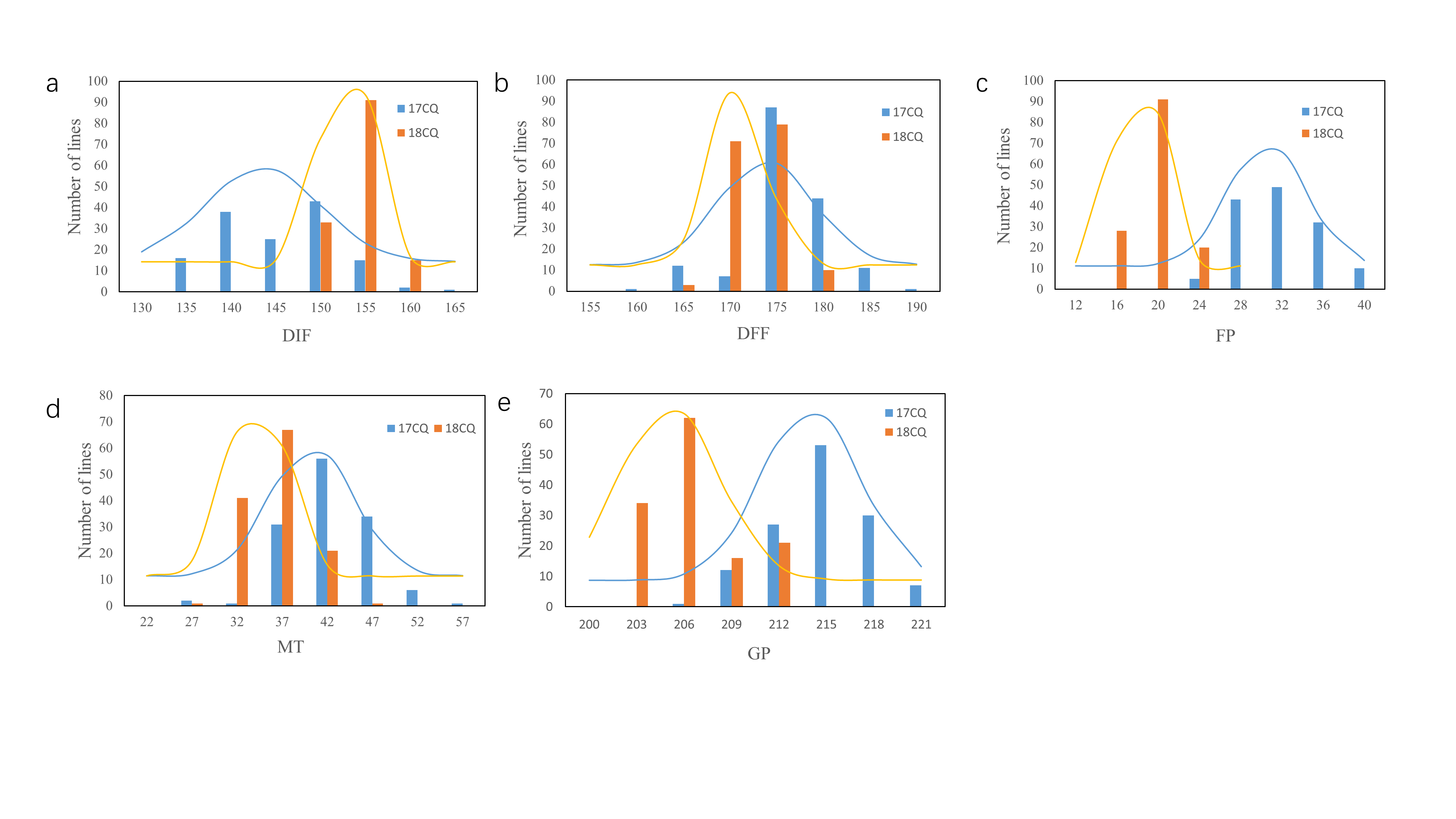

Supplement: Supplementary file 7 — Additional file 7: Fig. S3. Histogram of the frequency distribution of five growth period traits in RIL population in 2017 and 2018. [file 13068_2020_1774_MOESM7_ESM.tif]

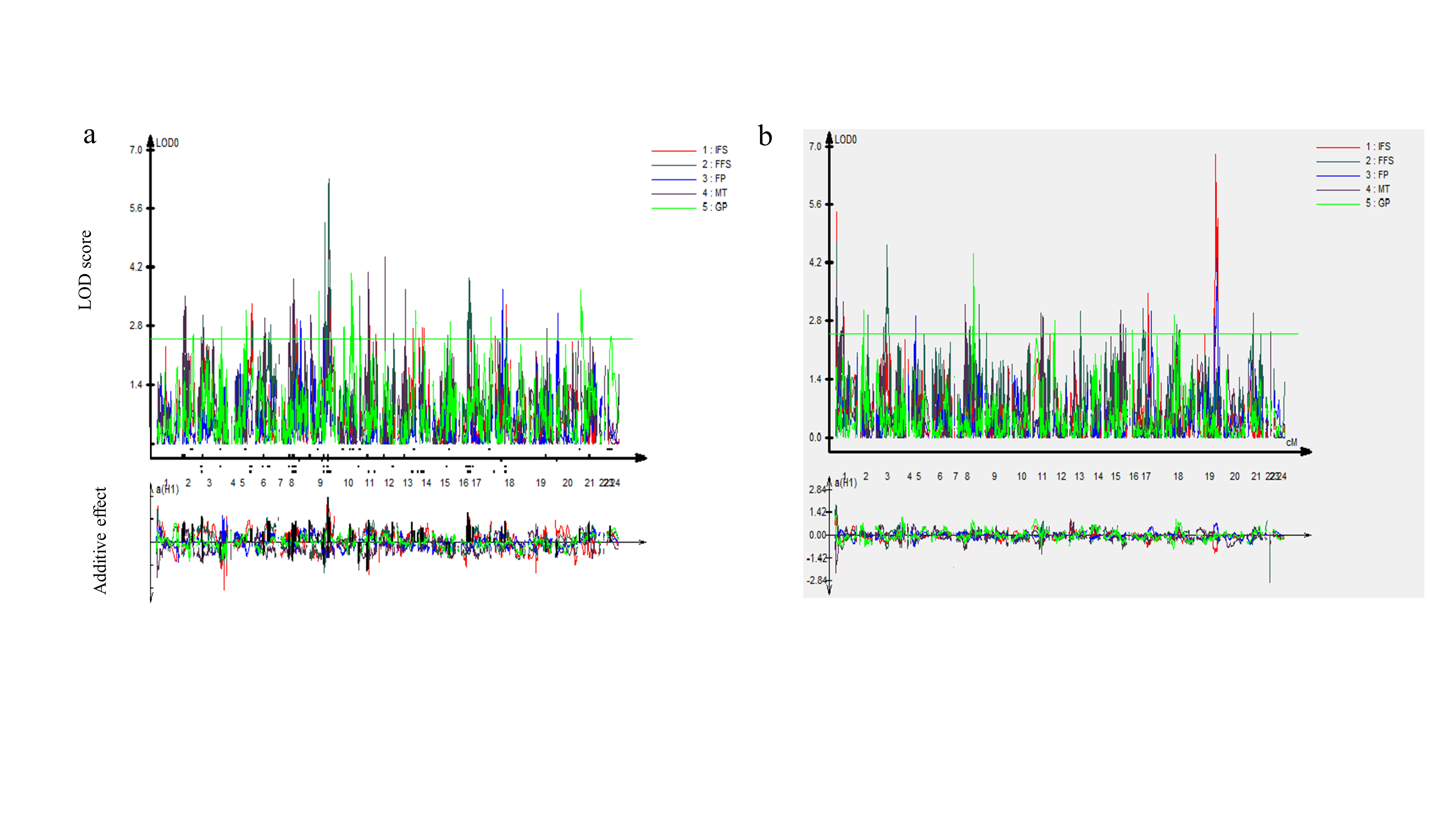

Supplement: Supplementary file 8 — Additional file 8: Fig. S4. The LOD score plots of five growth period traits across linkage groups in 2017(a) and 2018(b). Different traits are indicated by lines with various backgrounds (Red: QTL for initial flowering; green: the final flowering; blue: flowering period; black: maturity time; yellowish green: growth period). [file 13068_2020_1774_MOESM8_ESM.tif]

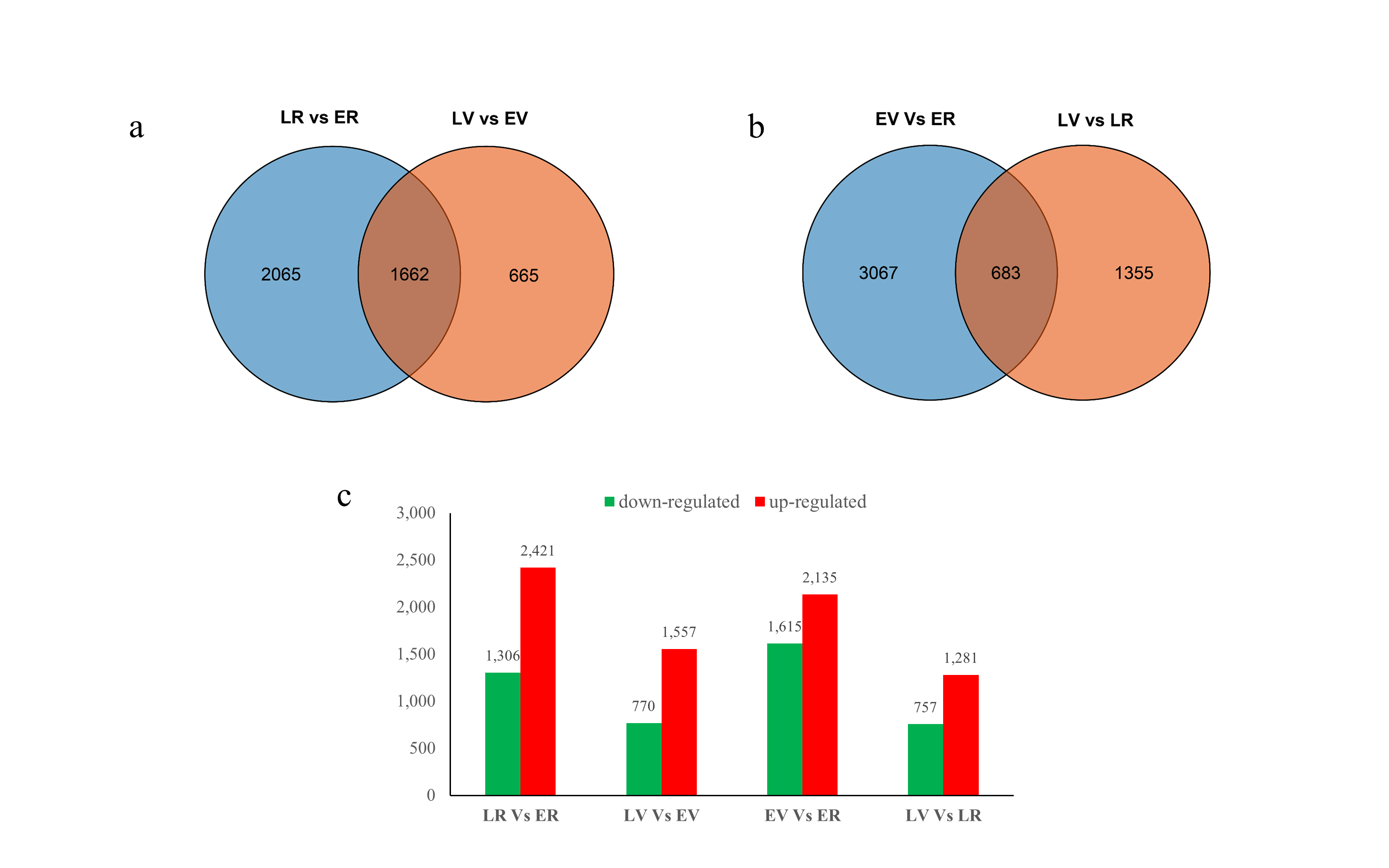

Supplement: Supplementary file 13 — Additional file 13: Fig. S5. The statistics of differential expression gene in four comparisons. a, the Venn diagram of DEGS identified in LR vs. ER and LV vs. EV; b, the Venn diagram of DEGS identified in EV vs. ER and LV vs. LR; c, the number of up-regulated and down-regulated genes identified in the comparisons of four groups. [file 13068_2020_1774_MOESM13_ESM.tif]

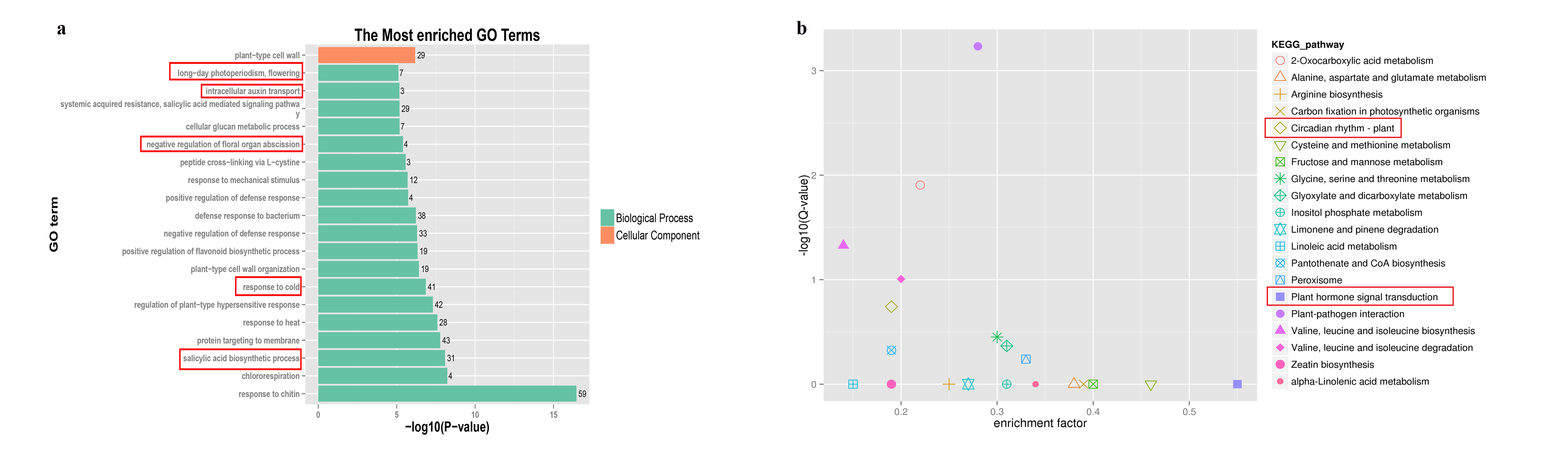

Supplement: Supplementary file 15 — Additional file 15: Fig. S6. Go and KEGG pathway significantly overrepresented of common genes between EV vs. ER and LV vs. LR. a, the enriched GO terms; b, the top 20 enriched pathways. [file 13068_2020_1774_MOESM15_ESM.tif]

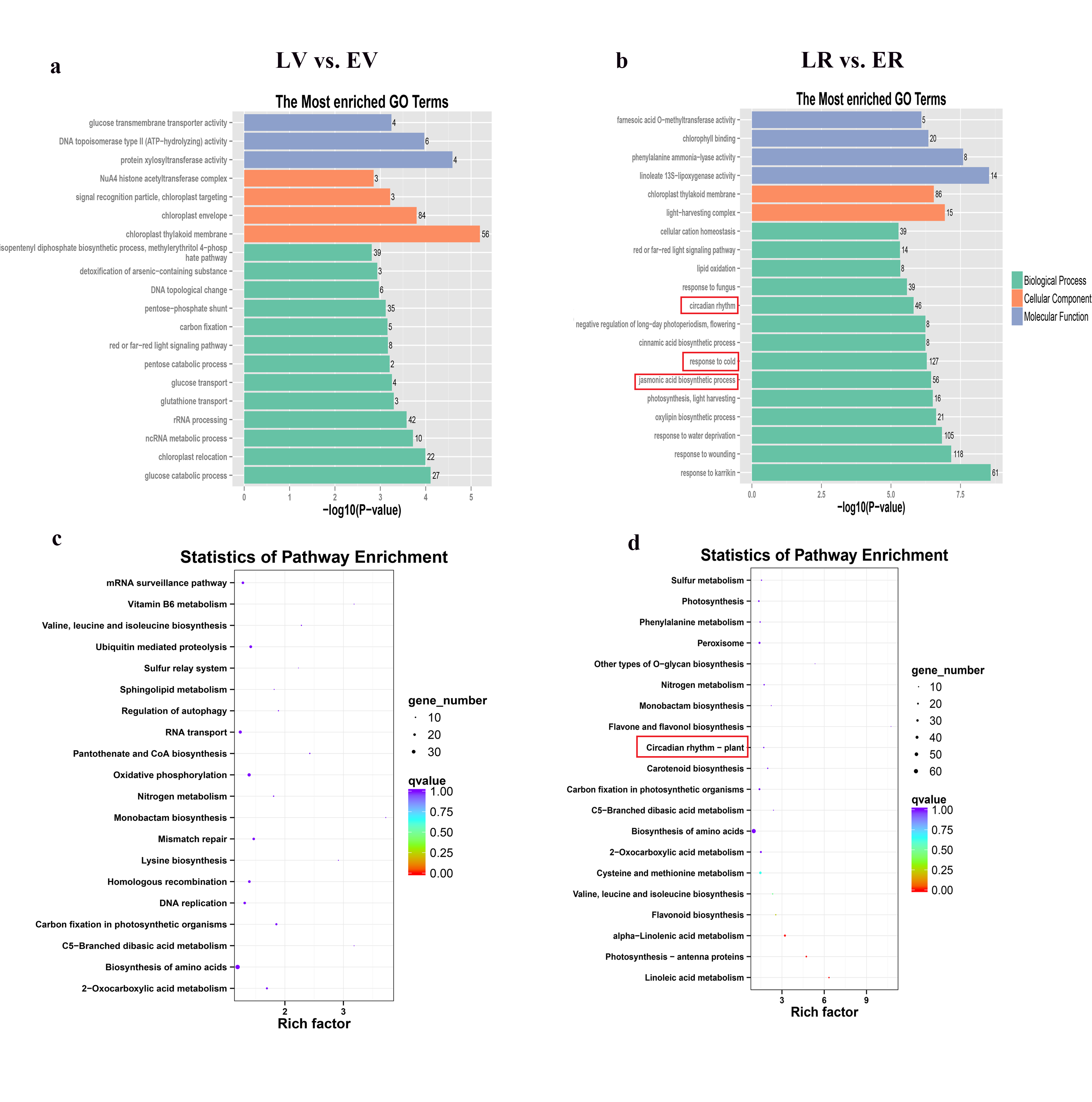

Supplement: Supplementary file 17 — Additional file 17: Fig. S7. Go terms and KEGG pathways enriched between the two extreme lines at each development stage. a, the enriched GO terms in LV vs. EV; b, the enriched GO terms in LR vs. ER; c, the top 20 pathways enriched in LV vs. EV; d, the top 20 pathways enriched in LR vs. ER. [file 13068_2020_1774_MOESM17_ESM.tif]

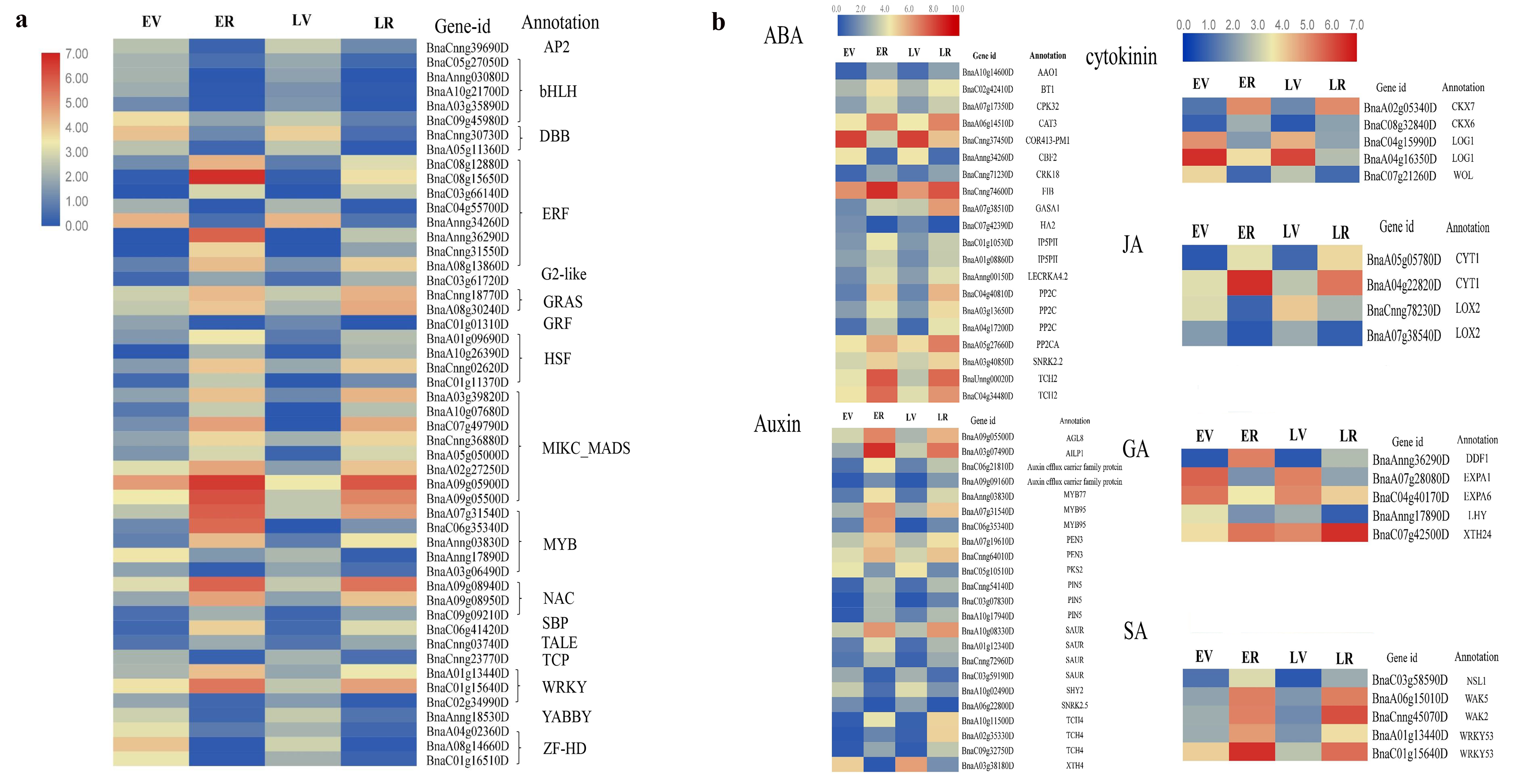

Supplement: Supplementary file 18 — Additional file 18: Fig. S8. Heatmap diagram of expression levels for DEGs involved in floral transition and flowering development-associated transcription factors (a) and phytohormone signaling pathways including ABA, auxin, JA, GA and SA (b). [file 13068_2020_1774_MOESM18_ESM.tif]

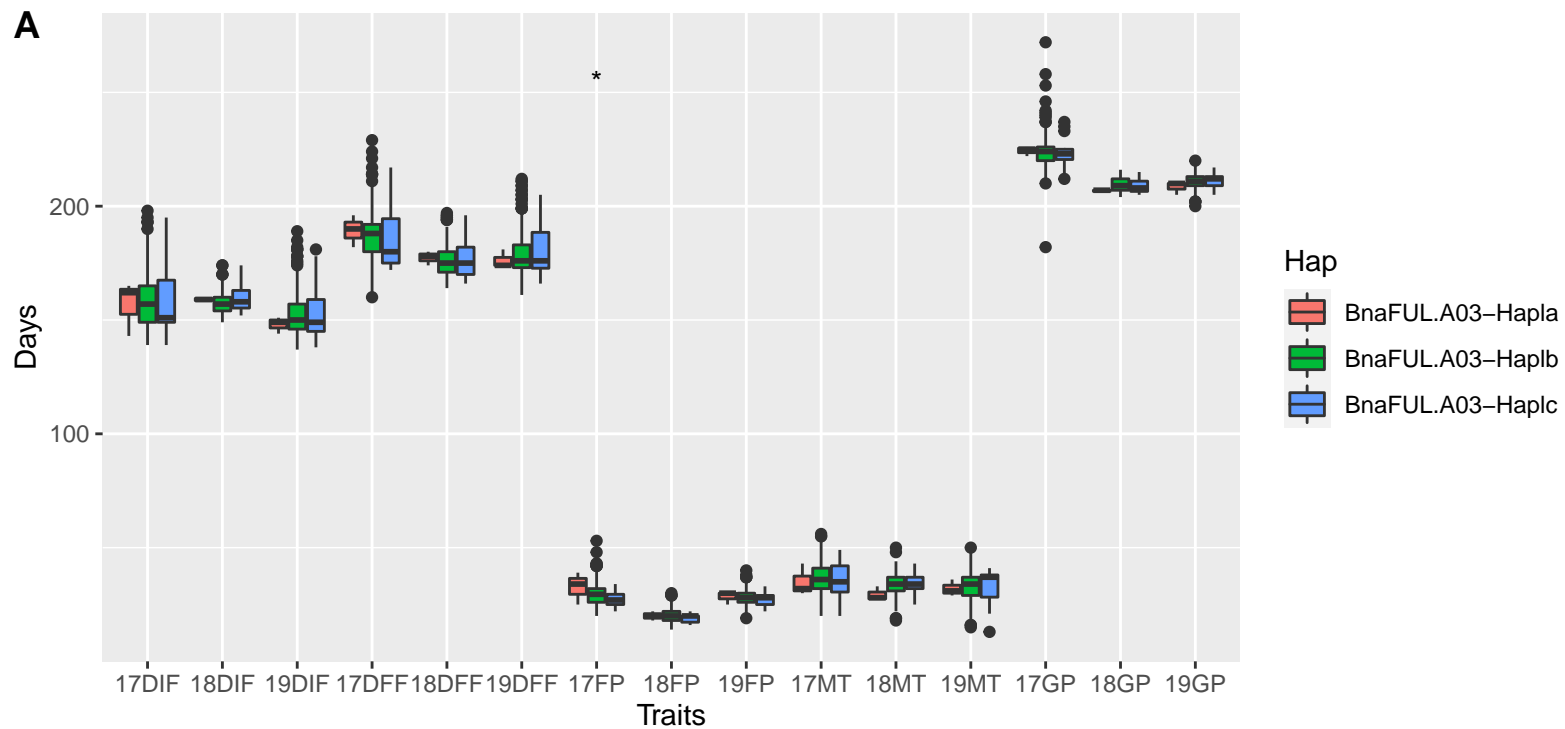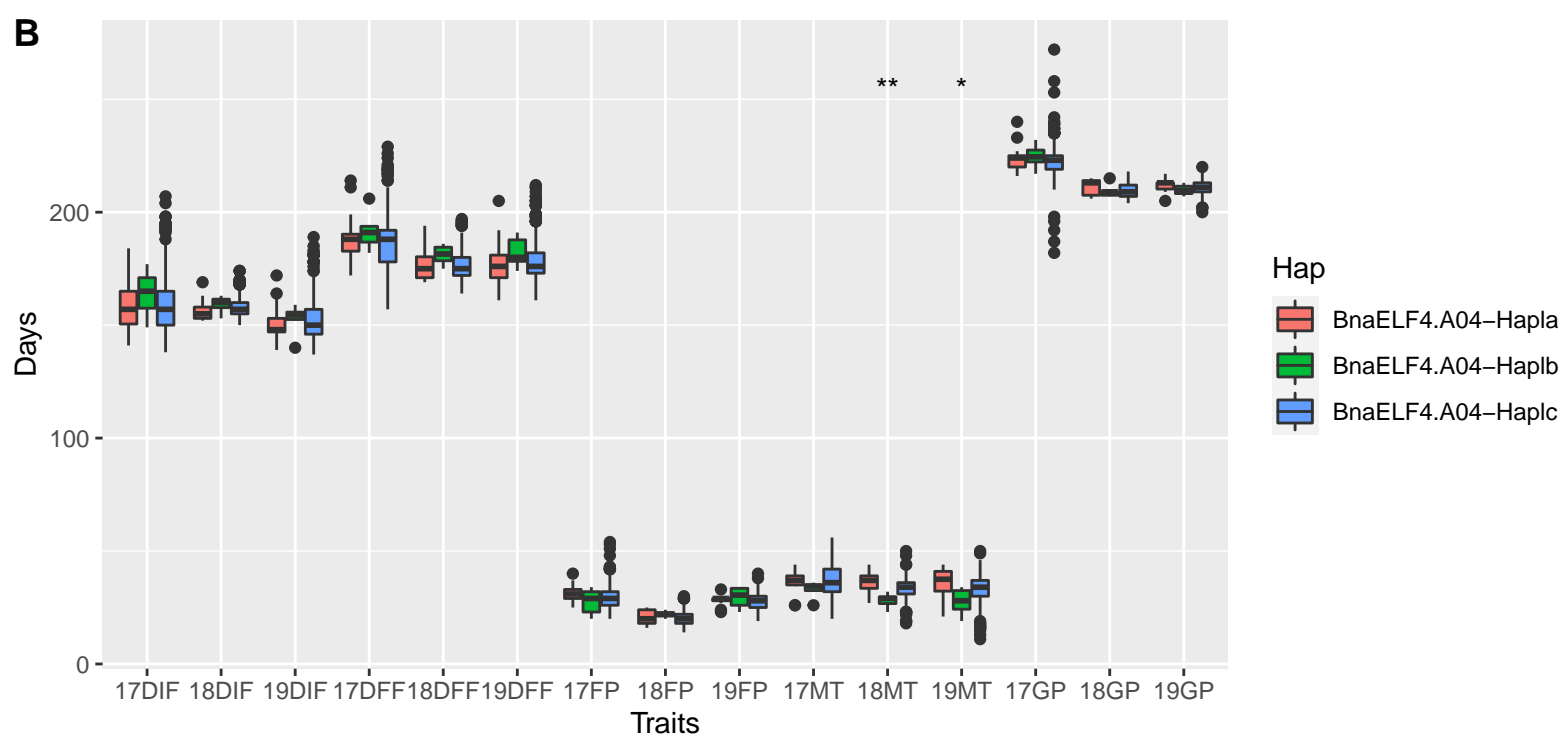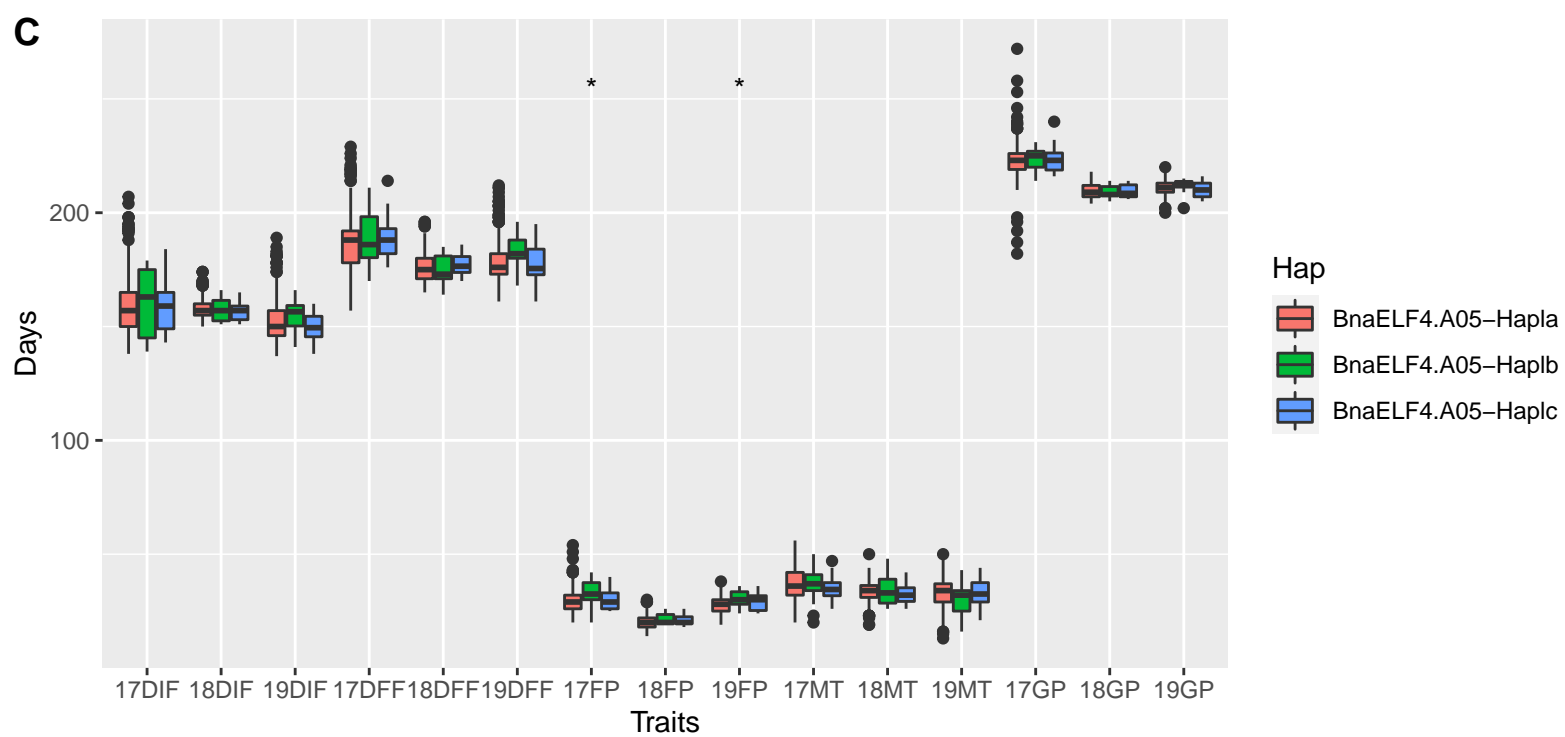

Supplement: Supplementary file 21 — Additional file 21: Fig. S9 The box-plot of different haplotypes of BnaFUL.A03, BnaELF4.A04 and BnaELF4.A05 on the basis of five growth periods in three years. [file 13068_2020_1774_MOESM21_ESM.pdf]

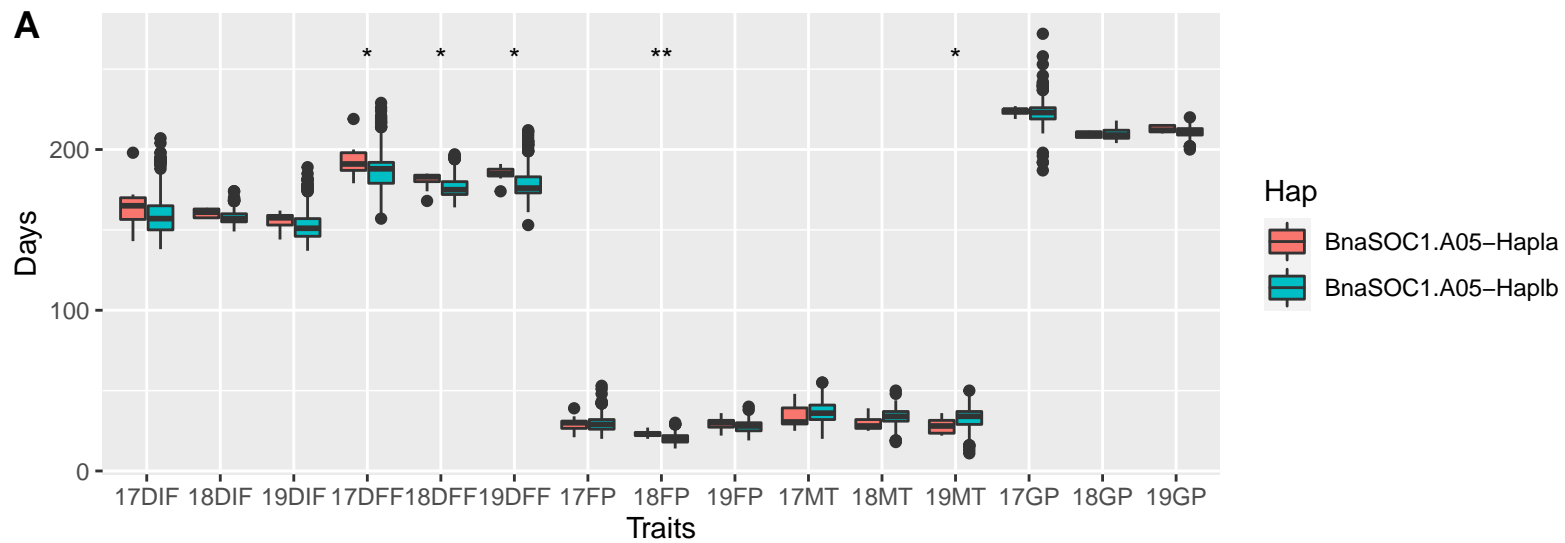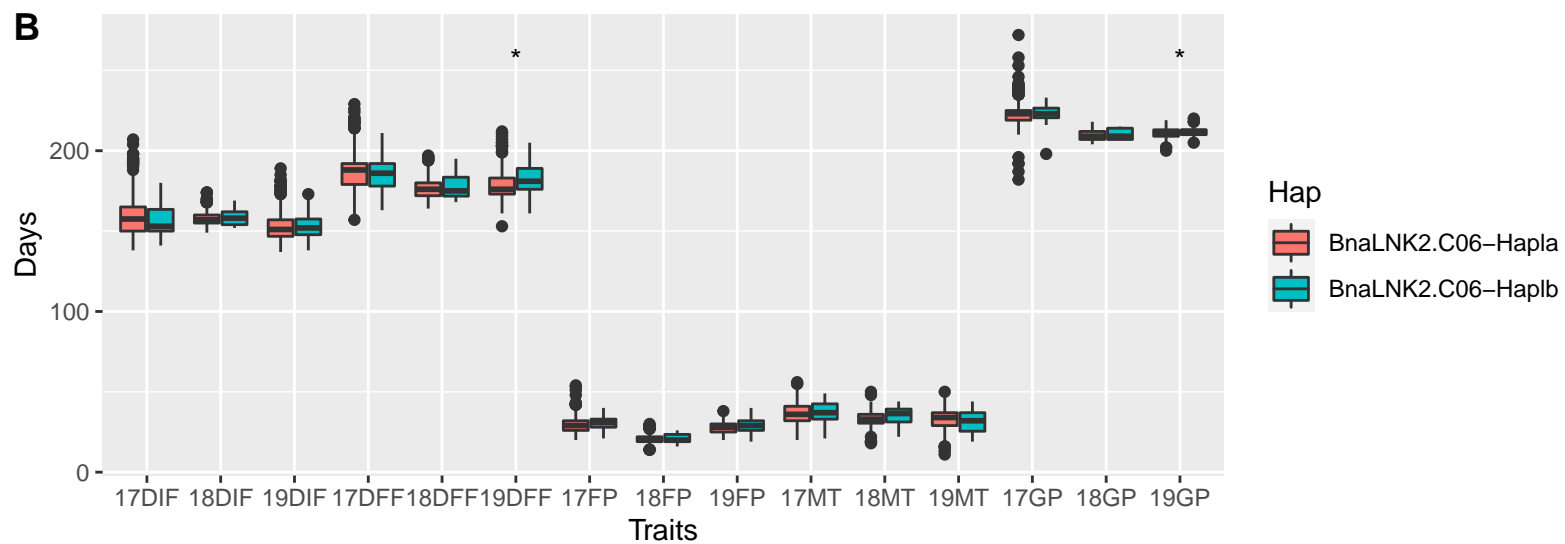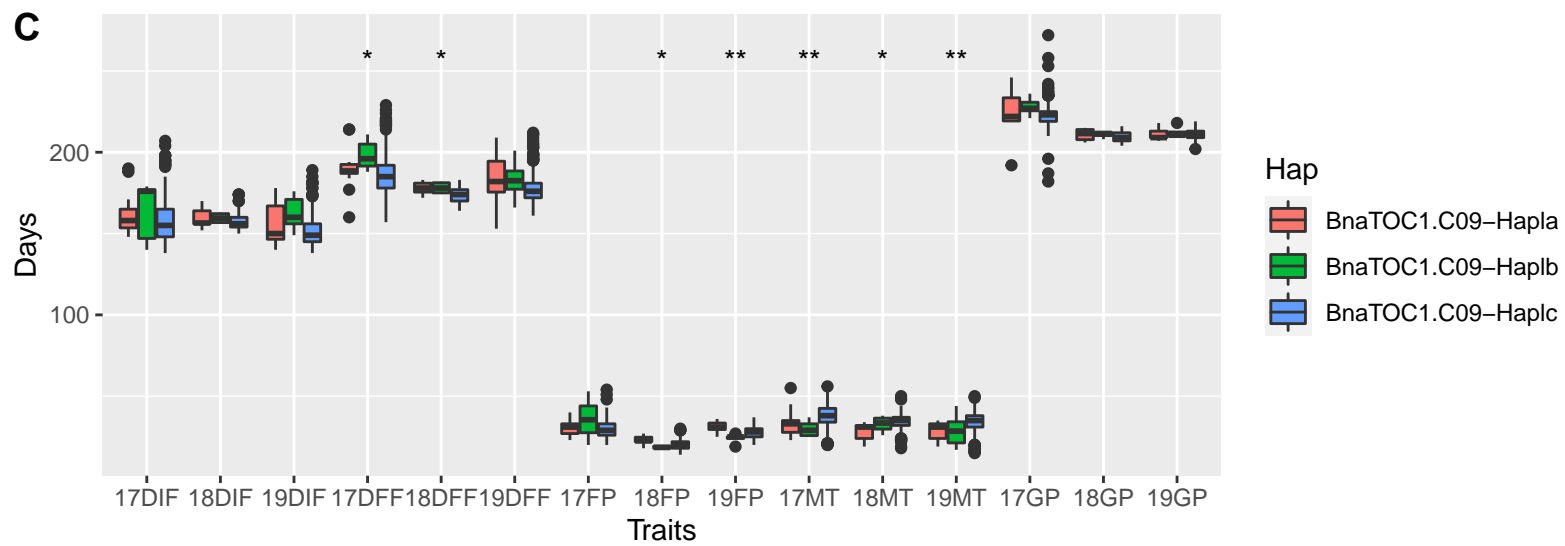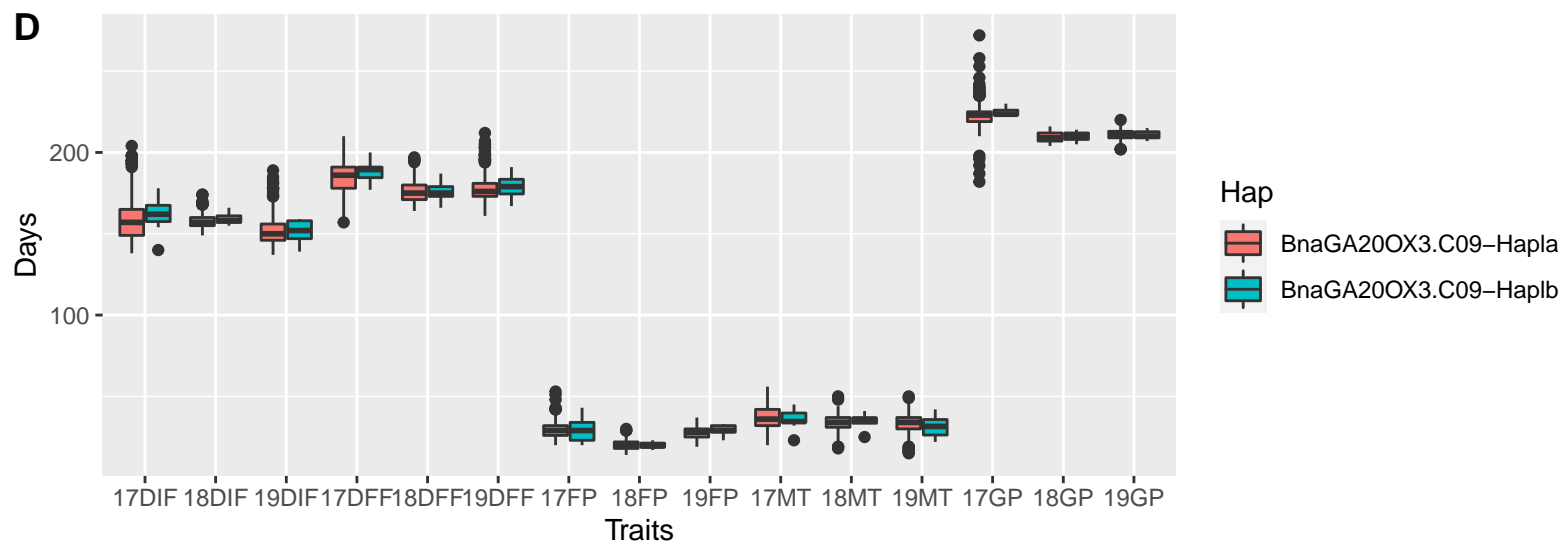

Supplement: Supplementary file 22 — Additional file 22: Fig. S10 The box-plot of different haplotypes of BnaSOC1.A05, BnaLNK2.C06, BnaTOC1.C09 and BnaGA20OX3.C09 on the basis of five growth periods in three years. [file 13068_2020_1774_MOESM22_ESM.pdf]

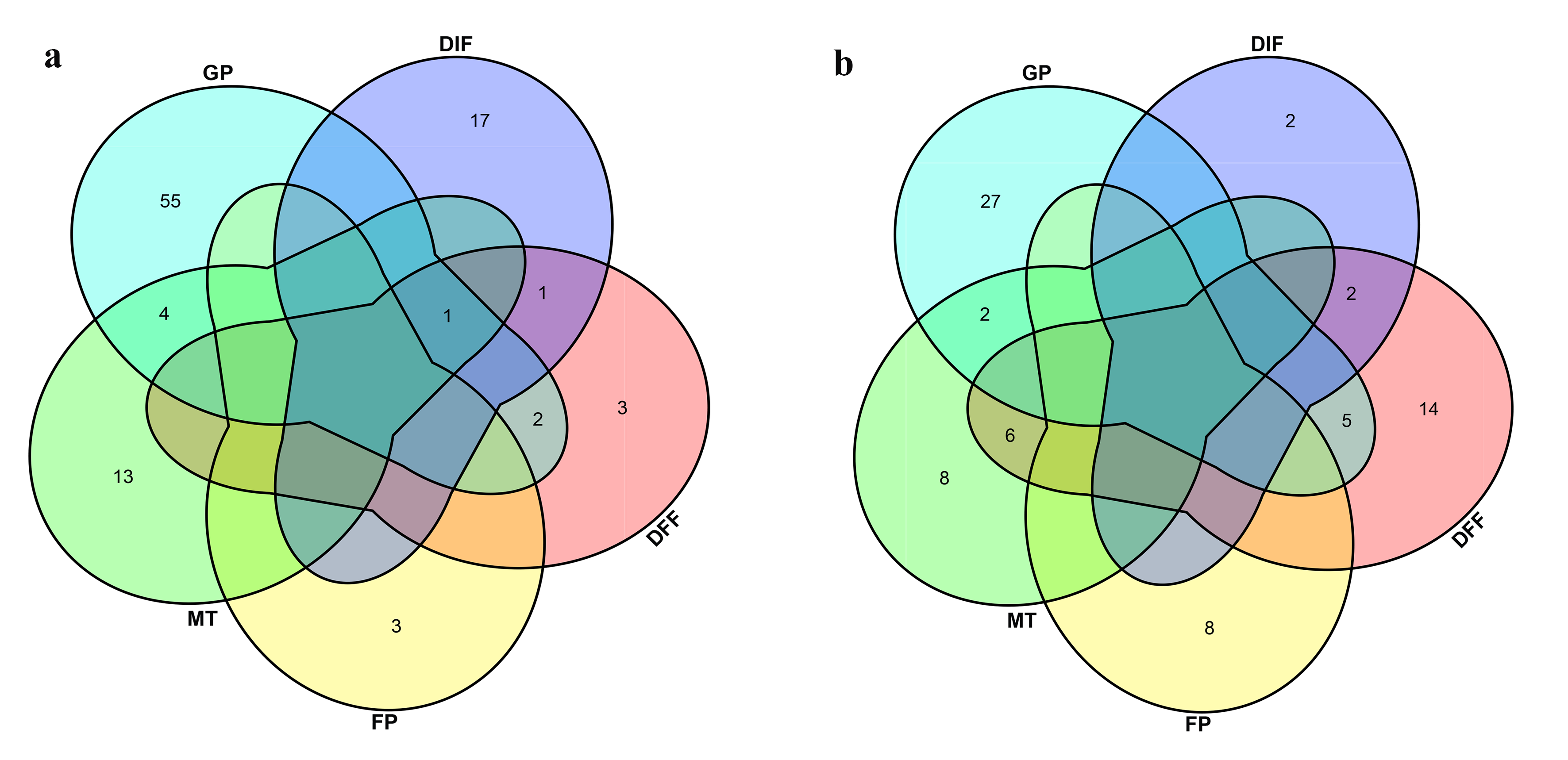

Supplement: Supplementary file 25 — Additional file 25: Fig. S11. The venn diagram of the number of flowering genes with pleiotropic effect identified in GWAS and linkage mapping. [file 13068_2020_1774_MOESM25_ESM.tif]
